# Infectious vaccine-derived rubella viruses emerge, persist, and evolve in cutaneous granulomas of children with primary immunodeficiencies

**DOI:** 10.1371/journal.ppat.1008080

**Published:** 2019-10-28

**Authors:** Ludmila Perelygina, Min-hsin Chen, Suganthi Suppiah, Adebola Adebayo, Emily Abernathy, Morna Dorsey, Lionel Bercovitch, Kenneth Paris, Kevin P. White, Alfons Krol, Julie Dhossche, Ivan Y. Torshin, Natalie Saini, Leszek J. Klimczak, Dmitry A. Gordenin, Andrey Zharkikh, Stanley Plotkin, Kathleen E. Sullivan, Joseph Icenogle

**Affiliations:** 1 Division of Viral Diseases, Centers for Disease Control and Prevention, Atlanta, Georgia, United States of America; 2 Department of Pediatrics, University of California, San Francisco, San Francisco, California, United States of America; 3 Department of Dermatology, Hasbro Children's Hospital and Warren Alpert Medical School of Brown University, Providence, Rhode Island, United States of America; 4 Division of Allergy and Immunology, Children's Hospital New Orleans, New Orleans, Louisiana, United States of America; 5 Department of Dermatology, Oregon Health & Science University, Portland, Oregon, United States of America; 6 Institute of Pharmacoinformatics, Federal Research Center “Computer Science and Control” of Russian Academy of Sciences, Dorodnicyn Computing Center, Moscow, Russian Federation; 7 Genome Integrity and Structural Biology Laboratory, National Institute of Environmental Health Sciences, US National Institutes of Health, Research Triangle Park, North Carolina, United States of America; 8 Integrative Bioinformatics Support Group, National Institute of Environmental Health Sciences, US National Institutes of Health, Research Triangle Park, North Carolina, United States of America; 9 Myriad Genetics, Inc., Salt Lake City, Utah, United States of America; 10 University of Pennsylvania Perelman School of Medicine, Philadelphia, Pennsylvania, United States of America; 11 Division of Allergy and Immunology, The Children’s Hospital of Philadelphia, Philadelphia, Pennsylvania, United States of America; University of Michigan, UNITED STATES

## Abstract

Rubella viruses (RV) have been found in an association with granulomas in children with primary immune deficiencies (PID). Here, we report the recovery and characterization of infectious immunodeficiency-related vaccine-derived rubella viruses (iVDRV) from diagnostic skin biopsies of four patients. Sequence evolution within PID hosts was studied by comparison of the complete genomic sequences of the iVDRVs with the genome of the vaccine virus RA27/3. The degree of divergence of each iVDRV correlated with the duration of persistence indicating continuous intrahost evolution. The evolution rates for synonymous and nonsynonymous substitutions were estimated to be 5.7 x 10^−3^ subs/site/year and 8.9 x 10^−4^ subs/site/year, respectively. Mutational spectra and signatures indicated a major role for APOBEC cytidine deaminases and a secondary role for ADAR adenosine deaminases in generating diversity of iVDRVs. The distributions of mutations across the genes and 3D hotspots for amino acid substitutions in the E1 glycoprotein identified regions that may be under positive selective pressure. Quasispecies diversity was higher in granulomas than in recovered infectious iVDRVs. Growth properties of iVDRVs were assessed in WI-38 fibroblast cultures. None of the iVDRV isolates showed complete reversion to wild type phenotype but the replicative and persistence characteristics of iVDRVs were different from those of the RA27/3 vaccine strain, making predictions of iVDRV transmissibility and teratogenicity difficult. However, detection of iVDRV RNA in nasopharyngeal specimen and poor neutralization of some iVDRV strains by sera from vaccinated persons suggests possible public health risks associated with iVDRV carriers. Detection of IgM antibody to RV in sera of two out of three patients may be a marker of virus persistence, potentially useful for identifying patients with iVDRV before development of lesions. Studies of the evolutionary dynamics of iVDRV during persistence will contribute to development of infection control strategies and antiviral therapies.

## Author summary

Primary immunodeficiency diseases (PID) are caused by genetic defects and lead to serious problems including chronic granulomas (abnormal collections (nodules) of inflammatory cells), sometimes lasting for decades and sometimes leading to severe ulcers. Initial reports (2014–2016), including our report of a blinded study using ultrasensitive virus detection in biopsies, proved the association between granuloma of the skin in PID patients and rubella virus. The viruses in these reports and the current report were derived from a widely used vaccine strain of the rubella virus. Work reported here shows that these vaccine-derived viruses are biologically different from the vaccine virus and that their genomes have changed. Genomic changes could be analyzed largely because the exact sequence of starting vaccine virus genome was known. These genomic differences are likely generated via mechanisms similar to those occurring during normal circulation of wild type rubella. We present data that newly recognized mechanisms for generation of sequence diversity in viruses (because of cellular deaminases) likely occurs in the generation of these vaccine-derived rubella viruses. Thousands of PID patients in the United States are likely shedding these vaccine-derived rubella viruses. Our work presented here characterizing viruses in diagnostic specimens highlights at least two areas where insufficient work has been done: 1) research on the properties of rubella virus (limited understanding of the antibody binding sites on the virus); 2) controlled research studies to assess the public health impact of viruses in populations with high immunity.

## Introduction

Rubella virus (RV) is an enveloped, single-stranded, positive-sense RNA virus in the *Rubivirus* genus, which has been recently moved from the *Togaviridae* to a new family, *Matonaviridae* [1]. A total of 13 RV genotypes, which represent 2 clades, have been recognized, but 2 genotypes, 1E and 2B, are currently the most common worldwide. RV replicates at low levels and produces little cytopathology both *in vitro* and *in vivo*. A distinct feature of RV is the ability to persist in the placenta and fetus and in immune privileged body sites of immunologically competent individuals [2, 3]. Persistent RV infection is associated with a congenital rubella syndrome (CRS) and a number of less common pathologies such as rubella encephalitis and Fuchs uveitis [4, 5]. The live attenuated vaccine strain, RA27/3 (a virus from the likely extinct 1a genotype and a part of the MMR vaccine), is currently used in the US and globally. It has high immunogenicity, generates long-term immunity after a single dose, is effective in preventing clinical disease, and has a very low rate of adverse events [6]. Worldwide, implementation of rubella vaccination programs has resulted in elimination of rubella and CRS from the Americas and significant reduction in the burden of disease in some developed countries [7]. Similar to wild type RV, RA27/3 can persist in immunologically competent individuals for a limited time causing mild complications, such as transient arthralgia or arthritis in adult women [8]. The vaccine virus involvement in the pathology of Fuchs uveitis is also suspected [5, 9]. The vaccine virus has not been associated with congenital defects, but asymptomatic persistent infections of the fetus have been reported after inadvertent vaccination of unknowingly pregnant women [10].

Primary immunodeficiency diseases (PID) are a group of hereditary disorders affecting different arms of the immune system [11]. PID patients usually have increased susceptibility to infections and have difficulties eliminating pathogens. Live vaccines, including rubella vaccine, are contraindicated for individuals with severe antibody deficiency, T-cell deficiencies or innate immune defects because they may cause severe or chronic disease [12]. Unfortunately, PID diagnosis often occurs after vaccination with MMR (usually given at the age of 12–15 months). Nevertheless, adverse outcomes related to MMR vaccination of children who are diagnosed with PID are thought to be rare [13].

Granuloma formation, a well-recognized disease in PID patients, is an accumulation of histiocytes and other immune cells near sites of chronic infection, which may persist for years sometimes resulting in significant pathology [14]. The estimated granuloma prevalence in PID patients is 1–4% and thus ~4,000 individuals in the US are expected to be affected [15]. RV antigen and RNA have been recently found in association with granulomas at various body sites (skin, liver, kidney, spleen, lung and bone periosteum) in children with a broad spectrum of PIDs [16–19]. RV positive cutaneous granulomas have been reported to develop 2–152 weeks (average 48 weeks) after MMR vaccination typically near the vaccination site, but can also appear at other body sites, e.g., face or legs, and then slowly spread [19]. Prominent T cell deficiencies, often with concurrent antibody deficiencies, are common characteristics of PID patients with RV positive granulomas [17, 19]. Immunohistochemical analysis of granulomatous lesions revealed that M2 macrophages in the center of granulomas most commonly harbored RV antigen [17]. Previously, mutated RA27/3 RNA was detected in a few cases but sequencing data were limited [16, 17]. As a result, little was known about the evolution of the vaccine virus during persistent infection in PID patients.

Our initial attempt to isolate infectious virus from the RV-positive skin granuloma of a single PID patient failed [17]. Accumulated deleterious mutations in the vaccine virus after a 22-year-long persistence in this case may have caused loss of infectivity of that virus. However, it was unclear whether loss of infectivity is a common feature of RA27/3-derived viruses within PID patients or a characteristic of vaccine virus evolution within that particular patient.

Here we report the isolation of infectious immunodeficiency-related vaccine-derived rubella viruses (iVDRV) from the skin biopsies of four PID patients collected at different times after vaccination. We have determined full genomic sequences of these iVDRV and characterized the changes relative to the parental RA27/3 virus with the objective of characterizing the RA27/3 evolution during persistent infection in PID patients. The replicative and persistence properties of the recovered iVDRV were compared with those of RA27/3 and wild type RV (wtRV) in WI-38, the primary human fibroblasts used to culture RA27/3 during attenuation [20]. This study also documents iVDRV detection in nasopharyngeal secretions raising the possibility of transmission of iVDRV strains to susceptible non-immune contacts.

## Results

### Virus isolation

Real time RT-qPCR analysis revealed variable amounts of RV RNA in the skin biopsies of four PID patients ranging from a total of about 5.6 x10^3^ to 1.7x10^5^ RV RNA copies per a biopsy sample (Table 1). No measles or mumps RNA were detected in these RNA samples by real-time RT-PCR, which is consistent with the results for the previously reported granuloma case [17]. Furthermore, only RV antigen was detected in the available tissue sections of cutaneous granulomas (LA, OR, and RI cases) by fluorescent immunohistochemical staining for measles and rubella antigens (S1 Fig). These data indicate that only rubella component of MMR vaccine persists in these four lesions.

**Table 1:** Patient and sample information.

Case ID	PID	Age at initial sampling (years)	Specimen type	Wks after initial sample	RV RNA amount ^a^	Virus isolation	Isolate name	Designation for genome sequence
CA	AT	10	skin biopsy	0	1.8x10^4^	Pos	CA6944	RVi/California.USA/43.16/GR RVs/California.USA/43.16/GR
			buccal swab	0	0	n.t.		
			buccal swab	0	0	n.t.		
			throat swab	8	0	n.t.		
			urine	8	0	n.t.		
			serum	8	0	n.t.		
			swab lesion 1	8	0	n.t.		
			swab lesion 2	8	0	n.t.		
RI	AT	17	skin biopsy	0	1.7x10^5^	Pos	RI6318	RVi/RhodeIsland.USA/9.17/GR RVs/RhodeIsland.USA/9.17/GR
			NP swab	0	0	n.t.		
			urine	0	0	n.t.		
			PBMC	0	0	Neg		
			serum	14	0	n.t.		
LA	AT	6	skin biopsy	0	5.6x10^3^	Pos	LA3331	RVi/Louisiana.USA/27.17/GR RVs/Louisiana.USA/27.17/GR
			throat swab	3	0	n.t.		
			urine	6	0	n.t.		
			serum	6	0	n.t.		
			NP swab	0	2.0x10^2^	Neg		RVs/Louisiana.USA/27.17/NP
			NP swab	3	3.7x10^2^	Neg		
			NP swab	9	0	n.t.		
			NP swab	12	0	n.t.		
			NP swab	15	0	n.t.		
OR	NBS	11	NP swab	0	0	n.t.		
			skin biopsy	6	1.2x10^4^	Pos	OR5810	RVi/Oregon.USA/05.18/GR RVs/Oregon.USA/05.18/GR

^a^—expressed as total RNA copies/entire skin biopsy or RNA copies/ml of NP swab sample

*Abbreviations*: AT—ataxia telangiectasia; NBS- Nijmegen breakage syndrome; NP–nasopharyngeal; n.t.—not tested (virus culture was not performed for RT-PCR negative samples).

The WHO-recommended protocol for RV isolation requires three passages (infection of fresh Vero cells monolayers with media from a previous, week-long, passage) because of typically low RV quantities in clinical samples. The failure to isolate infectious virus in the previously reported case prompted us to modify the culture protocol to enhance virus recovery from biopsy specimens (see Methods). To estimate the number of infected cells in the cultures, cells from each isolation were seeded onto chamber slides after each passage and then immunostained for RV structural proteins E1, E2 and C. Almost all cells were positive in the 14-dpi cultures of CA6499 and RI6318, whereas less than 1% RV-positive cells were detected in the 21-dpi cultures of the LA3331 and OR5810. This suggests the presence of low quantities of infectious virus in the granulomas of the LA and OR cases and/or the reduced abilities of the recovered viruses to infect and/or spread in Vero cell monolayers. Infectious rubella viruses were harvested after 14 days (CA and RI cases) or 21 days (LA and OR cases) of culture in Vero cells; the isolate designations are indicated in Table 1. Taken together, these data suggest that infectious viruses with distinctive growth properties are present in the lesions of these four patients.

### Virus shedding

The presence of infectious virus in biopsies led us to assess virus shedding in urine samples, NP swabs, buccal swabs and lesion scrapings by first testing for RV RNA by real-time RT-qPCR. All samples were negative except the two sequential NP swabs taken three weeks apart from the LA case patient (Table 1). The RV RNA concentration in both NP swabs was low, ~2-4x10^2^ copies/ml of the swab sample. Virus culture using 0.5 ml of both samples (~100 RV RNA molecules/flask) was unsuccessful most probably due to the low number of infectious particles in the sample. The ratio of foci forming units (ffu) per RV genome was previously determined to be about 1/40 in both RA27/3 and wtRV-infected cell cultures [21] and was lower in clinical samples. Thus we estimate only 2–3 ffu per flask or less in these two isolation attempts. The presence of RV RNA in the sequential NP samples indicates that viral shedding into a nasopharyngeal cavity can occur. Three sera and one PBMC sample from three patients were all negative by RT-qPCR indicating a lack of viremia.

### Sequence analysis of the iVDRVs genomes

**Fig 1:**
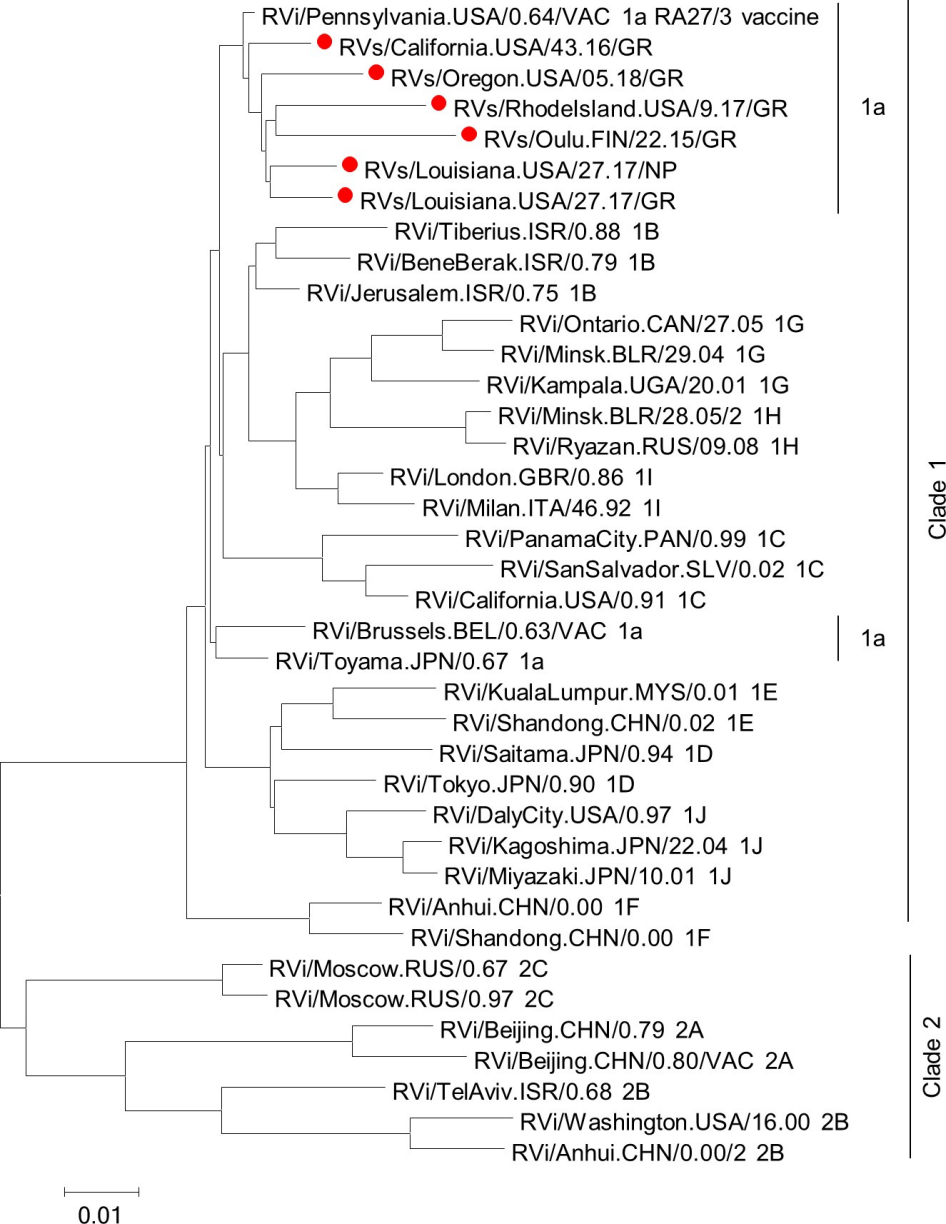
Phylogenetic tree of iVDRV. The genetic relationships between the consensus genome sequences from each original granuloma sample and the whole genomes of the WHO reference viruses were inferred using the Maximum Likelihood method in MEGA7. All taxa are labeled with WHO names with iVDRV sequences marked with red dots. The genetic distances were computed using the Maximum Composite Likelihood method. The scale bar indicates the number of base substitutions per site. RA27/3 and iVDRVs represent a separate branch on the tree with RA27/3 being basal.

Full genome sequences were obtained from granuloma biopsy specimens (RVs) and passage 1 (P1) virus isolates (RVi) by Sanger sequencing of overlapping RT-PCR products. The P1 CA6944 isolate was passaged three more times to obtain a high titer P4 virus stock. The consensus sequences of the full RV genomes in the P1 and P4 stocks were identical showing consensus sequence stability for at least three passages in Vero cells. Phylogenetic analysis of the sequences obtained from primary granuloma samples (including previously described RVs/Oulu.FIN/22.15/GR (GeneBank#KU958641.1), with full genomes of the WHO RV reference viruses showed that all sequences derived from the patients’ samples belong to genotype 1a (Fig 1). RA27/3 vaccine strain was basal to all sequenced iVDRV. These viruses were somewhat more distantly related to other vaccine strains, which are also genotype 1a.

**Fig 2:**
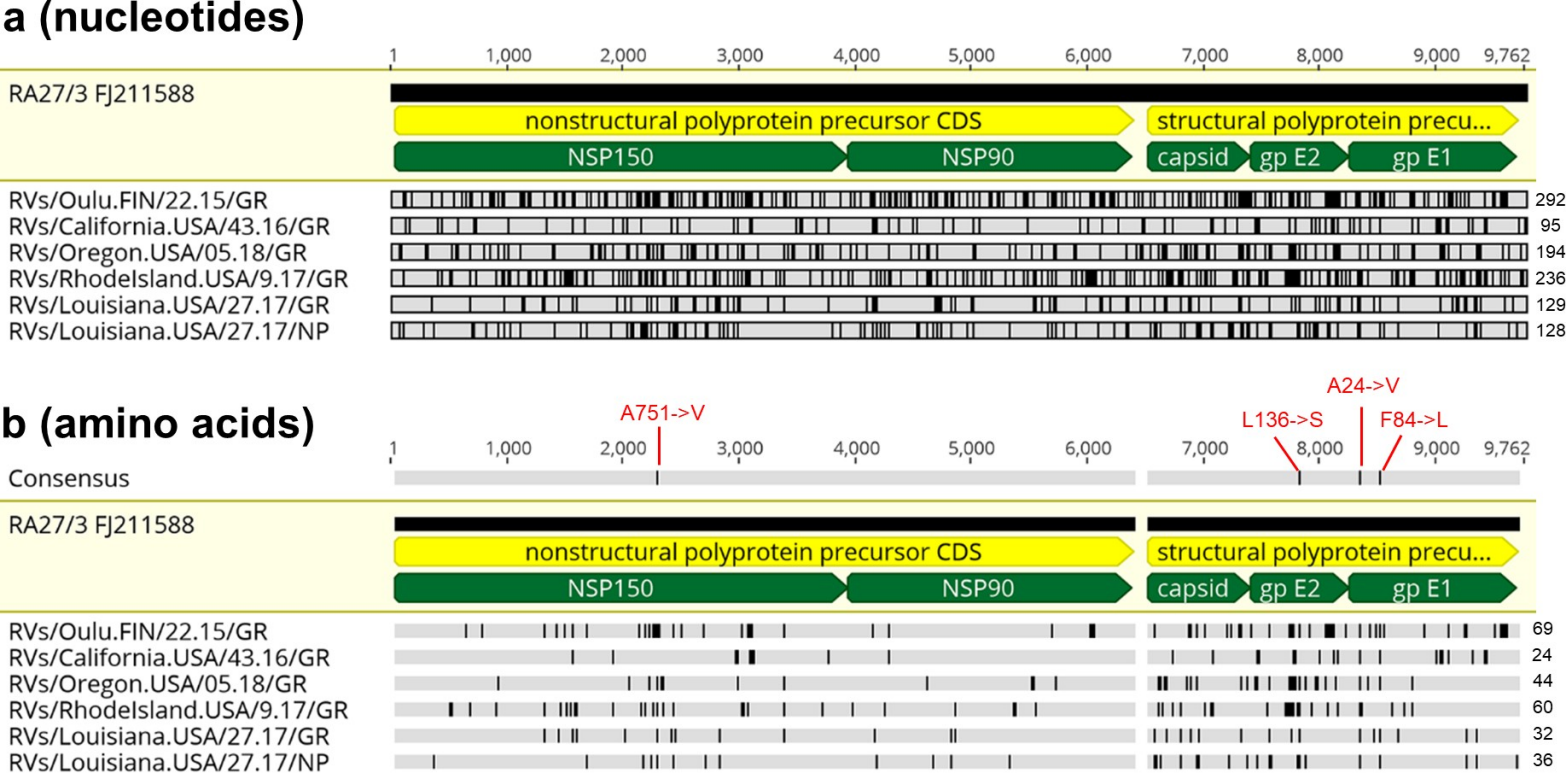
Diversity of iVDRV sequences. **a.** Nucleotide and **b.** amino acid substitutions in iVDRV genomes relative to parental RA27/3 virus (GenBank #FJ211588) are depicted as vertical lines. The total number of substitutions are shown for each iVDRV on the right side. The consensus in **b.** represents amino acids identical in >50% sequences with the shared substitutions shown in red above the consensus; the indicated positions in the substitutions were the positions in the corresponding RV proteins. The positions of the coding sequences (CDS) and proteins are indicated by dark yellow and green pointed bars, respectively. The reference sequence is indicated by light yellow shading. The figure was prepared with Geneious software.

Comparative analysis of six iVDRV RVs and four RVi genomic sequences revealed multiple (from 95 to 292) single nucleotide substitutions compared to the RA27/3 parental virus, many of them nonsynonymous (Fig 2, S1 Table, S1 Data). Amino acid substitutions in the iVDRV proteins were observed at a total 247 positions (S2 Table). The majority of substitutions (192 out of total 247, 76.5%) were found only in iVDRV strains, whereas the remaining substitutions in 55 amino acid positions (23.5%) were also observed in various wtRV strains and may represent reversions to wtRV. Notably, 142 substitutions (57.5%) occurred at invariant amino acids in wtRV, which circulated worldwide during a period 1961–2012 (S2 Table, S2 and S3 Data). Thus, the spectrum of amino acid substitutions appears to be different between wtRV, which circulated in normal populations during 1961–2012, and iVDRV persisting in immunodeficient individuals (S2 Table), but additional studies of cross sectional diversity are needed to further confirm this observation.

### iVDRV quasispecies

**Fig 3:**
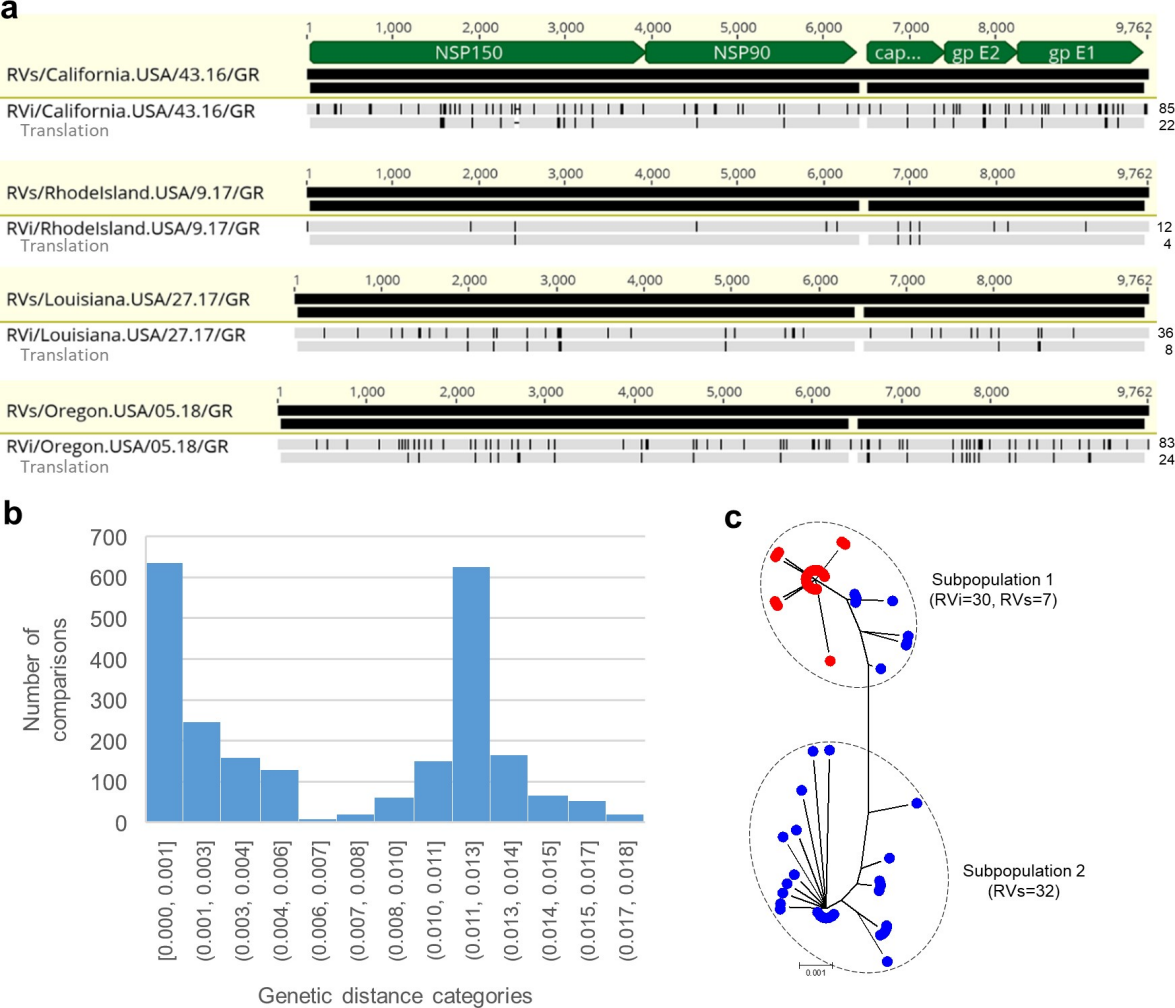
iVDRV quasispecies in tissue and viral isolate. **a.** Nucleotide and amino acid substitutions in consensus RVi sequences relative to paired consensus RVs sequences shown by vertical lines for each case. The reference RVs sequences are indicated by light yellow shading. The number of substitutions are shown for each RVi on the right. The regions in the genomic sequence which encode proteins are indicated by the green pointed bars. **b.** Distribution of pairwise genetic distances between individual quasispecies within primary granuloma sample and the virus isolate from the CA patient. Each bar in the binned histogram represents the number of comparisons in each distance class. Note, the distance categories are not identical due to rounding. Underlying data can be found in the S4 Data file. **c.** Neighbor-joining tree (non-rooted) for quasispecies within the granuloma sample (n = 39, blue circles) and virus isolate (n = 30, red circles) from the CA case. The genetic distances were computed using the Maximum Composite Likelihood method. The scale bar indicates the number of base substitutions per site.

The consensus RVi genomic sequences of four isolates were different from the paired RVs sequences derived directly from granuloma biopsies (Fig 3A). This difference is presumably because many viruses in the patients’ granulomas do not enter and/or replicate in Vero cells. Additionally, double peaks occurred at multiple positions in the RVs chromatograms, indicating the presence of mixed nucleotides. Such sequence ambiguities were minimal in the RVi chromatograms. These data show the existence of different populations of viruses (quasispecies) in tissues and clinical isolates.

Attempts in our and other labs to use next generation sequencing techniques for deep sequencing of RV genomes directly from clinical samples have been unsuccessful so far; sequencing depth rarely exceeded 10, probably because of low quantities of viral RNA in clinical samples, the high GC content of the RV genome, and the strong genomic secondary structure [16, 22]. Hence we characterized viral diversity by molecular cloning followed by Sanger sequencing. We selected the granuloma biopsy from the CA case and paired virus stock for the comparison of RV genetic variability in an original clinical sample versus virus isolate because of the largest differences between RVi and RVs consensus sequences: a 6-nt (2-aa) deletion and a total of 85-nt substitutions in RVi relative to RVs, of which 22 were nonsynonymous. The E1 gene fragment was amplified with high fidelity DNA polymerase using total RNA isolated from the granuloma biopsy or from the CA6944 P1 viral stock, cloned and sequenced. In total, 39 RVs and 30 RVi clones were sequenced and analyzed for diversity and complexity. Genetic diversity within quasispecies in the original granuloma specimen (mean and max genetic distance = 0.006 and 0.018) was higher than in the P1 virus stock (mean and max genetic distance = 0.001 and 0.003) (S4 Data). Low complexity was observed in the virus stock (18/30 (60%) identical sequences), whereas iVDRV quasispecies in the granuloma were highly heterogeneous. We found two peaks in the distribution of the pairwise genetic distances between individual quasispecies (Fig 3B, S4 Data). Within-patient phylogenetic analysis of the quasispecies further confirmed that two distinct iVDRV subpopulations were present in the granuloma lesion (Fig 3C). All quasispecies from the virus stock grouped together with only 7 (18%) quasispecies from the lesion in subpopulation 1, which explains the difference between the consensus sequences for RVs and RVi in this case. There was only one synonymous substitution in the quasispecies of the isolate compared to the consensus sequence of quasispecies in the granuloma subpopulation 1. Taken together, these data demonstrate a complex population structure of iVDRV quasispecies in the granuloma lesions with only a small subset of quasispecies being infectious in Vero cell culture.

### iVDRV sequence evolution within patients

**Fig 4:**
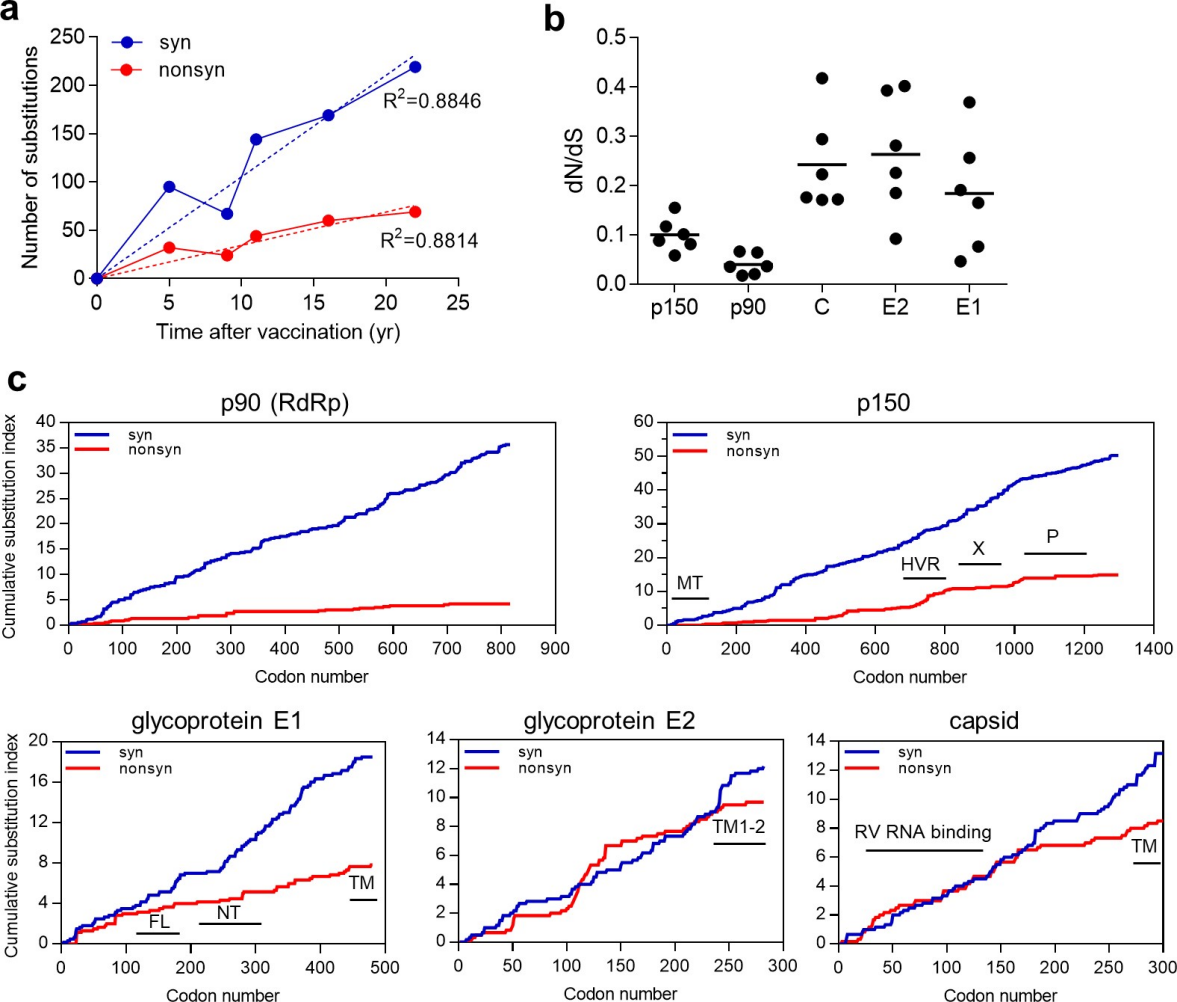
Analysis of iVDRV evolution patterns. **a.** Relationship between the number of synonymous and nonsynonymous substitutions in the consensus genomic sequences directly from the specimens (RVs) and time elapsed after vaccination. **b.** Sequence divergence of the individual genes by a patient. dN/dS ratios were calculated with the SNAP tool. **c.** Plots of cumulative behavior of the average number of synonymous and nonsynonymous substitutions per a codon (cumulative substitution index) across each gene were calculated with the SNAP tool. Underlying data for Fig 4C can be found in the S5 Data file. The following domains are indicated: MT—methyltransferase, HVR -hypervariable region, X—ADP-ribose binding, P—protease, FL—fusion loops I and II, NT—neutralizing epitopes, TM—transmembrane domains. Note the NT domain is actually composed of four separate epitopes.

The relationship between the number of synonymous and nonsynonymous substitutions in RVs genomic sequences from each case and the time after vaccination is presented in Fig 4A. A constant increase in the number of substitutions per a RV genome with time indicates continuous sequence evolution of iVDRV in PID patients. The overall trend is a positive linear association between the number of synonymous and nonsynonymous substitutions and the duration of persistence and, thus, either or both substitution types may be useful as molecular clocks for the determination of the time iVDRV genomes have persisted in patients.

Since the sequence of vaccine virus and the time of vaccine administration were known, the rate of evolution of iVDRV strains was calculated by direct pairwise comparisons of iVDRV RVs genomic sequences with the sequence of the ancestor, RA27/3 vaccine, using SNAP. The estimated iVDRV evolution rates varied slightly among the patients (S3 Table) with the average dS rate of 5.7 x 10^−3^ subs/site/year and average dN rate of 8.9 x 10^−4^ subs/site/year. The dS rates were similar for all genes, while the dN rates differed among the genes. The overall iVDRV evolution rate was estimated to be 1.8 x 10^−3^ subs/site/year.

**Fig 5:**
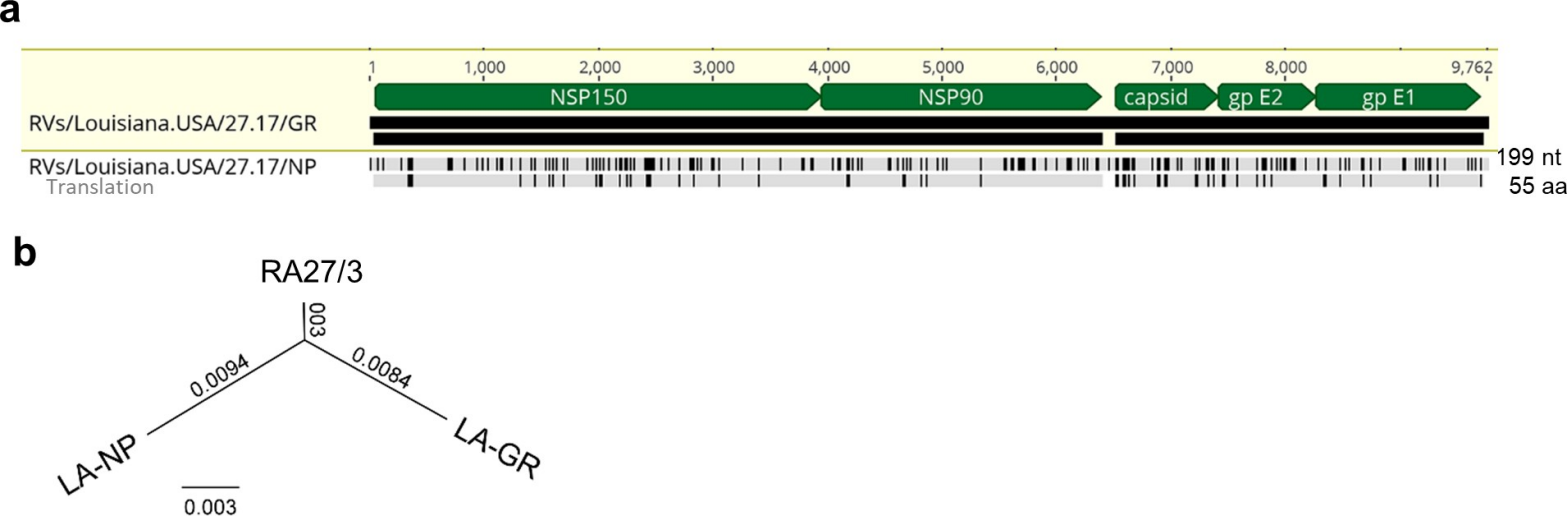
Different iVDRV lineages in different body sites (arm skin and NP). **a.** Divergence of the iVDRV sequence in the NP fluids relative to iVDRV in the skin granuloma. Nucleotide and amino acid substitutions in the NP-derived sequence relative to granuloma-derived sequence (shaded in light yellow) are depicted as vertical lines. The number of substitutions are shown at the right side. The positions of encoded proteins in the genomic sequence are indicated by green pointed bars. **b**. Neighbor-Joining phylogenetic tree showing the genetic relationships between the whole genome sequences of RA27/3, LA-NP and LA-GR viruses. The genetic distances were computed using the Maximum Composite Likelihood method. The scale bar indicates the number of base substitutions per site.

The consensus genomic sequences and protein sequences of iVDRV from anatomically separated body sites (arm skin and NP cavity) of the LA case were substantially different from each other (Fig 5A) indicating coexistence of different iVDRV lineages in this patient. Phylogenetic tree (Fig 5B) shows the genetic relationships between full genomes of RA27/3 and two lineages, LA-GR and LA-NP. Using the determined dS and dN rates, we estimated that two lineages separated ~1.1 years or 1.0 year, respectfully, after MMR.

The synonymous substitutions were distributed evenly throughout the genomes while the nonsynonymous substitutions occurred predominately in the genes encoding structural proteins and the hypervariable regions in the p150 gene (Fig 2). To evaluate the selection pressure on individual RV genes, we calculated a dN/dS ratio for each RV gene for each patient (Fig 4B). Calculation of the dN/dS ratio is one of the means to estimate the presence and direction of selection [23]. Overall, the SP genes were less constrained and more diverged than the NSP genes. Since different functional and structural domains in proteins are often under a different selection pressure and evolve at different rates [24], we examined evolutionary patterns in the iVDRV proteins by calculating the cumulative behavior of the average dN and dS per codon, moving codon by codon along each gene (Fig 4C, S5 Data). The dS curves were similar between the different genes and along the each gene length representing a baseline of mutation fixation because synonymous mutations are largely neutral. The increase in cumulative substitution index, however, differed substantially between the genes and different regions of the same gene. The regions with constant cumulative dN values indicate strong purifying selection and, mostly, define conserved protein domains, e.g. the methyltransferase, ADP-ribose binding and protease domains of the p150 gene and the entire p90 gene encoding RNA-dependent RNA polymerase (RdRp). Glycoprotein E1 is the most conserved among the SP proteins; all domains appear to be under strong negative pressure except the first 100 residues. Similar dN and dS curves in the N-terminal half of the capsid (C) protein suggest neutral evolution in this region, which is responsible for association with genomic RV RNA [25]. The C-terminal part of C protein appears to be under the purifying selection, but the function of this domain is not yet clear. The C protein has been shown to enhance RV replication, interact with a number of cellular proteins and inhibit apoptosis, but specific amino acids/domains involved in those interactions have not been determined [26, 27]. Evolution of most of the E2 protein appears to be neutral since dN and dS curves were similar. Alternatively, E2 protein could be a subject to diversifying and purifying selection. The E2 region between aa 110 and 200, where non-synonymous mutations predominate, could be under positive selection. Unfortunately, functional and structural domains in E2 have not been defined in detail.

### Analysis of signatures of RNA-editing enzymes

**Fig 6:**
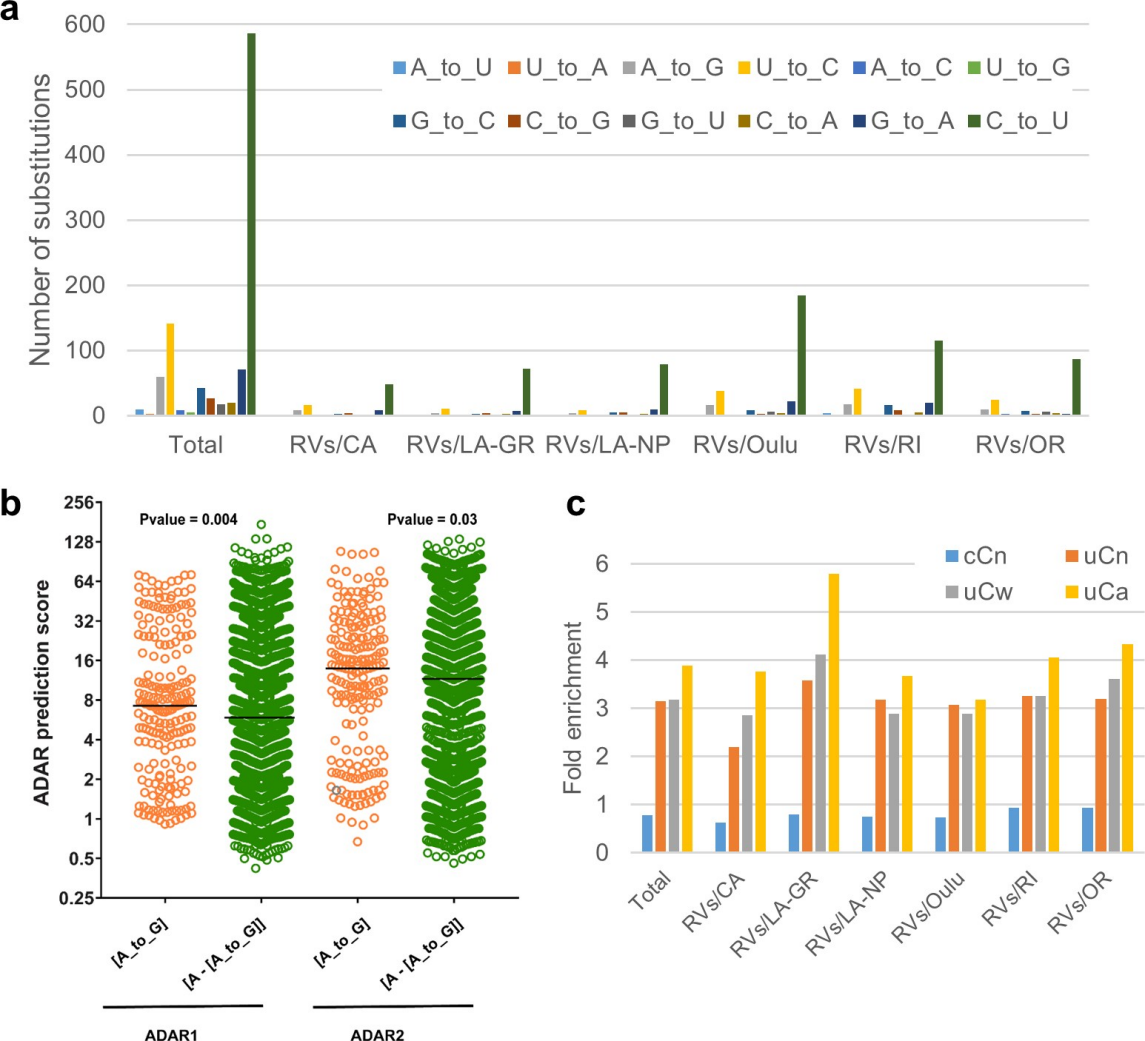
RNA editing signatures in iVDRVs. **a.** Spectra of single-nucleotide substitutions in iVDRV RVs genomes. Underlying data for Fig 6A–6C can be found in the S6 Data file. **b.** ADAR1 and ADAR2 prediction scores for adenines. The scores were calculated for 60 [A to_G] mutations in the positive strand and for 141 [A_to_G] mutations in the negative strand of the viral genome, as well as for the adenines that mutated to bases other than guanines along with adenines that did not mutate at all ([A–[A_to_G]]) (Note: Since, by convention, all mutations in S6 Data file are listed as changes in the positive strand, the negative strand [A to G] mutations are presented in this Table as [U to C] changes in the positive strand). The y axis denotes the prediction scores of hADAR1 and hADAR2 activity expressed as log2. The black horizontal line in the graphs denotes the median value of the prediction scores. P-values are shown above the scatter plots. **c**. Enrichments with APOBEC editing motifs in iVDRV RVs genomes. Mutated nucleotides are shown in capital letters within trinucleotide mutation motifs. Enrichment values were calculated for C to U mutations induced in the RV positive strand as described in Methods. P-values of one-sided Fisher’s exact test calculated as described in [37] can be found in the tab “Fisher_test_Fig6C” of S6 Data file.

We detected clear preference for C-to-U changes (expressed as changes in the positive RNA strand) over all other base substitutions in iVDRV RVs consensus sequences (Fig 6A, S6 Data). The second major component of the mutation spectrum was U-to-C substitutions. There was smaller number of C-to-U and U-to-C changes in the RV negative strand than in the positive strand (expressed as G-to-A and A-to-G changes in the positive strand in the Fig 6A). The mutation profile and the strand bias agree with the previously suggested roles of APOBEC (apolipoprotein B mRNA editing enzyme, catalytic polypeptide-like) cytidine deaminases and ADAR (Adenosine Deaminase Acting on RNA) in generating nucleotide diversity in RNA viruses [28–30].

We used the web-based tool InosinePredict to generate prediction scores for ADAR1 and for ADAR2 mutagenesis in each adenosine of the iVDRV RVs genomic sequences. In support of these enzymes playing a role in mutating adenines in the viral DNA, the prediction scores for positions containing A to G mutations exceeded prediction scores for adenines in positions that were not mutated or contained mutations other than A to G (Fig 6B, S6 Data).

Most of mutations were in cytosines of the viral positive strand (Fig 6A). The capability for cytidine deamination in RNA was well established for APOBEC1, while APOBEC 3 enzymes are commonly viewed as acting exclusively in DNA [31]. However, there are recent reports suggesting that the APOBEC3 subfamily might also act on pathogen and host RNA in certain types of cells [32–35]. The preferred motif for APOBEC3G deamination is cCn (where n is any nucleotide; mutated nucleotide capitalized), while the other members of the APOBEC3 family as well as APOBEC1 are characterized by a preference for tCn motif with higher preference for tCw (w = A or T) [29, 36]. The diagnostic signature for APOBEC3A and APOBEC3B was narrowed to tCa trinucleotide [37]. Therefore, we explored enrichment with these mutation signatures in C-to-U mutations induced in the positive strand of six iVDRV RVs sequences.

Interestingly, in each iVDRV strain as well as in the total mutation catalogue, there was depletion of cCn motif characteristic of APOBEC3G. However, there was strong enrichment with tCn-specific signatures, which constituted around 30% of all mutations in the positive strand. Splitting tCn signature into sub-components that have even higher preference to APOBEC enzymes revealed that enrichment with tCa signature exceeded the enrichment with either tCn or with tCw (Fig 6C, S6 Data). All enrichments with tCn, tCw and tCa signatures were highly statistically significant with P-values of one-sided Fisher’s exact test = < 0.01 in each case (see the tab “Fisher_test_Fig6C” of S6 Data file). This indicates that one or more t(u)Cn-specific APOBECs play a role in generating diversity in iVDRV populations. Additional studies are also needed to understand connections between the dynamics of viral RNA secondary structure and the relative impacts of APOBEC and ADAR editing activities, which act on to mutually excluding substrates–single stranded and double stranded states in viral RNA folds.

### Structural context of amino acid substitutions in E1 protein

Many amino acid changes occurred in E1, E2, and C proteins, but interpretation of their functional significance is limited by what is known about functional domains in the RV structural proteins. The E1 glycoprotein plays a crucial role in RV infectivity by mediating receptor binding and membrane fusion [38, 39]. E1 is the better immunogen than E2 and C and four neutralizing epitopes were mapped to this protein [40–42]. At least one weak neutralizing epitope of E2 was identified but not precisely mapped [43]. Less is known about rubella-specific MHC class I restricted CTL responses and only three CD8+ T cell epitopes have been identified so far (two of them overlap), all located in the capsid protein [44, 45]. Changes in RV neutralizing B cell epitopes and CD8+ T cell epitopes were detected in all cases except the RI case (Table 2).

**Table 2:** Substitutions in B and T cell epitopes in iVDRVs.

Epitope	Sequence in RA27/3	Substitutions in iVDRV					
		RVs FIN	RVs/RVi CA	RVs/RVi RI	RVs/RVi LA	RVs LA(NP)	RVs/RVi OR
Neutralizing B cell epitopes							
NT1:E1_221-239_	LGSPNCHGPDWASPCQRHS	-	-	-	-	-	-
NT2:E1_245-251_	**L**VGATPE*	-	L245V	-	-	-	-
NT3:E1_260-266_	**A**DDPLLR	-	A260V	-	-	-	-
NT4:E1_274-285_	VWVTPV**I**G**S**QAR	I280T	I280M S282A	-	-	-	I280T
CD8+ T cell epitopes							
C_9-22_	MEDLQKALE**T**QS**RA**	T18A	-	-	R21C	A22I	-
C_11-29_	DLQKALE**T**QS**RA**LR**A**EL**A**A	T18A	-	-	R21C	A22I A25E A28V	-
C_264-272_	RI**ET**RSARH	T267I	-	-	-	E266G	-

*Mutated residues in the epitopes are shown in bold.

**Fig 7:**
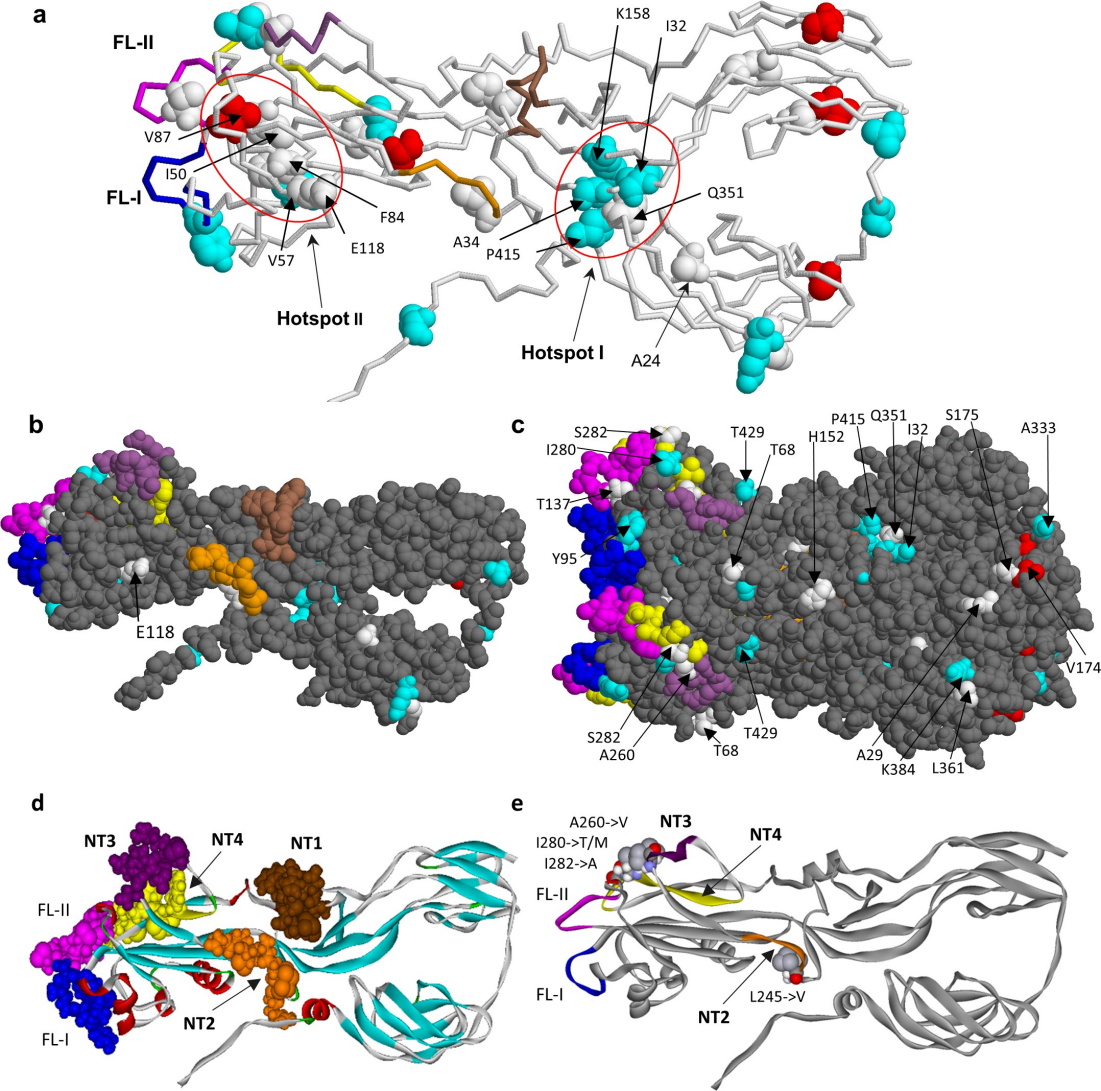
Location of amino acid substitutions in 3D structure of E1 glycoprotein. **a.** E1 monomer with all the identified substitutions mapped. The models of mutations were based on the coordinates from PDB entry 4adg. Mutational hotspots are shown encircled by a red line, the hotspot I including the residues I32, D34, K158, Q351, P415 and the hotspot II including the residues I50, V57, F84, V87, E118. The mutation A24->V is common in all iVDRV strains studied. The mutation F84->L was found in 5 of 6 strains and is located in the vicinity of the fusion loops. The color codes used: the variant residues found both in RVs and RVi (white spacefill), only in RVs (cyan spacefill), only in RVi (red spacefill); neutralizing epitopes NT1 (residues 225–235, brown), NT2 (245–251, orange), NT3 (260–266, violet) and NT4 (274–285, yellow); fusion loops (FL-I, residues 89–98, blue; FL-II, residues 130–138, magenta). **b, c.** Mutated amino acid residues on the surface of E1 monomer (**b**) and trimer (**c**) in iVDRVs (solvent accessibility >0.5, probe size 1.4A). **d**. The NT epitopes in E1 monomer. **e**. Possible escape mutations in the E1 neutralizing epitopes NT2, NT3 and NT4.

Since data on the antigenic structure of E2 and C proteins are limited, the detailed analysis of the mutations was only performed for E1. The three dimensional structure of the M33 RV strain E1 has been recently solved [46]. The amino acid sequences of E1 glycoprotein from M33 and RA27/3 strains (both belong to the genotype 1a) are identical. To gain insight into the driving forces of E1 evolution within PID patients, we mapped 39 amino acid substitutions, which occurred in RVs and RVi, onto the spatial structure of a single chain of E1 (Fig 7, S4 Table). Analysis revealed two “mutation hotspots” in the E1 spatial structure, which were defined as clusters of commonly mutated residues in van der Waals contact with one another. The mutation hotspot I involves residues I32, A34, K158, Q351, P415 in the vicinity of the neutralizing epitopes NT1 and NT2. None of the hotspot I substitutions (except Q351) were present in infectious virus isolates suggesting these mutations may be detrimental for RV infectivity in cell culture. The mutation hotspot II, which includes hydrophobic residues I50, V57, F84, V87 and the residue E118, is located near the E1 fusion loops. There were two substitutions in E1 shared by iVDRV: F84 reversion to wt L84 occurred in five out of six iVDRV and A24V substitution, which occurred in all six iVDRVs but not in wtRV strains (S2 Table). Many mutated residues (23 out of total 39) are exposed on the E1 surface (Fig 7B and 7C, S4 Table) and, thus, could be involved in direct interactions with host molecules.

Several substitutions occurred in E1 neutralizing epitopes NT2-NT4 in three iVDRV strains, while NT1 epitope was invariant (Table 2). A260V substitution in the NT3 epitope and two mutations in the NT4 epitope, S282A and I280T/M, were located in the proposed membrane contact region of E1 in close proximity to the fusion loops [46]. The substitutions in aa position 280 (the NT3 epitope) co-occurred in three iVDRV strains and, thus, might be important for interactions with neutralizing antibody. The spatial location of the NT3 and NT4 epitopes near the fusion loops suggests that antibodies targeting these epitopes may neutralize RV infectivity by interfering with the fusion process thus preventing cell entry. L245V substitution was mapped to the NT2 epitope, which is located in close proximity to the NT1 epitope and similar to the NT1 is not exposed on the surface of the trimer. The neutralization mechanism of antibodies targeting NT2 is mostly likely similar to the mechanism proposed for NT1, which is interference with the formation of E1 trimer, which is a pre-fusion form of E1 [46]. Taken together, these data suggest that granuloma-associated rubella virus is under selective pressure from both neutralizing antibodies and T cells.

### Growth properties of iVDRV strains

The replication and persistence properties of RA27/3 vaccine and clinical isolates form acute RV infections are substantially different [21]. To test if vaccine-derived viruses in granulomas have changed from the vaccine phenotype, we compared the growth properties of four iVDRV strains with growth properties of RA27/3 and RV-Dz in WI-38 human fetal fibroblasts, the primary cell culture used for RA27/3 attenuation [47]. RV-Dz is a well-characterized wild type strain of genotype 1E [48].

**Fig 8:**
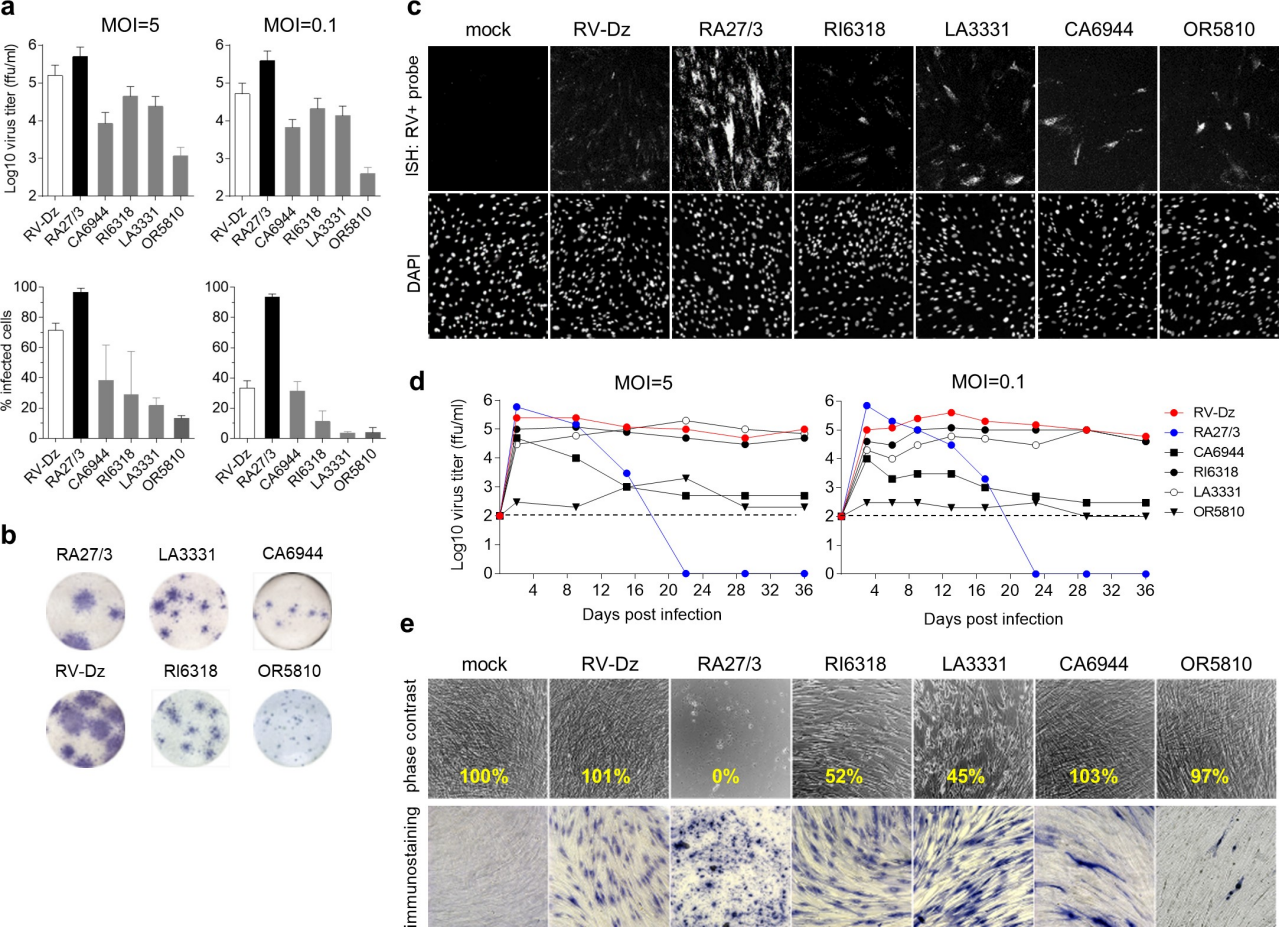
iVDRV growth properties. **a.** Virus yields and percentages of RV-positive cells after infection of WI-38 with iVDRV, wtRV and RA27/3 strains at MOI = 5 (2 dpi) and MOI = 0.1 (3 dpi). Virus titers in the media were determined by titration on Vero cells, the number of infected cells was estimated by immunostaining for E1 protein. Data are presented as a mean +/- s.d. (n = 3, each experiment was performed in duplicate). **b.** Foci of infection of iVDRV isolates on Vero cells in comparison to wt and vaccine foci revealed by immunostaining for E1 at 6 dpi. **c.** Representative images from two independent experiments showing the results of the RNA-FISH for positive-strand RV RNA in WI-38 mock infected or infected with RA27/3 or RV-Dz at MOI = 5 (2 dpi). Nuclei were counterstained with DAPI. **d**. iVDRV persistence in WI-38. Growth curves of the indicated strains were shown for MOI = 5 and 0.1. Media were collected every 3–6 days and the extracellular viruses were titered on Vero cells. The representative results of two independent experiments each done in duplicate are shown. Limit of the assay detection (1x10^2^ ffu/ml) is depicted by the dashed line. **e.** Phase contrast images of mock infected or infected (MOI = 5) cells at 36 dpi. Note cytopathic effects of RI6318 and LA3331 and the lack of live cells in RA27/3-infected wells. The adherent cells from one of two duplicate wells at 36 dpi were counted; the percentage of remaining adherent cells in each well was calculated relative to the mock infected well (yellow text). The cells in the second duplicate well were immunostained for E1.

To compare virus yields and percentage of infected cells, cell monolayers were infected with each virus strain at high (5 ffu/cell) and low (0.1 ffu/cell) multiplicity of infection (MOI). High MOI allows comparing efficiency of virus entry and replication while low MOI provides information on efficiency of cell-to-cell spread. At both MOIs after 2–3 days, RA27/3 infected almost the entire WI-38 monolayers, producing an infectious titer of 5x10^5^ ffu/ml, while replication of RV-Dz was less effective (Fig 8A). In contrast, iVDRV infected fewer cells in the monolayers (5–40%) producing 1–2.5 logs less infectious virus depending on the isolate. Similar to RV-Dz, iVDRV spread after low MOI infections was reduced compared to RA27/3. The size of foci formed by iVDRV on Vero monolayers were smaller than that of RV-Dz and RA27/3, confirming reduced abilities of iVDRVs to spread in cell culture (Fig 8B). The efficiencies of cell spread varied depending on the iVDRV isolate with OR5810 being the least efficient.

RV RNA replication on the single cell level was assessed using RNA-FISH. WI-38 cells were mock infected or infected with the RV strains at MOI = 5 for two days and then genomic RV RNA was detected by *in situ* hybridization with a probe set specific for the positive RNA strand (Fig 8C). As previously observed in endothelial cells [21], the total amounts of genomic RV RNA per a cell were substantially higher in RA27/3 than RV-Dz infected WI-38 cells. In iVDRV infected monolayers, most cells produced low quantities of genomic RV RNA, similar to that of RV-Dz, but isolated cells producing somewhat higher levels of cellular RV RNA than RV-Dz were also observed (Fig 8C).

The abilities of the iVDRV isolates to persist in WI-38 cells were compared after high and low MOIs. Virus titers in the medium and cytopathic effects (CPE) was monitored for 36 days. No substantial differences were seen in persistence characteristics after different MOIs. After the initial peak of replication, starting at day 2, RA27/3 began inducing cell death, which resulted in complete destruction of the monolayers by 15 dpi (MOI = 5) and 17 dpi (MOI = 0.1) (Fig 8D and 8E). In contrast, RV-Dz persisted at the same level (~10^5^ ffu/ml) for the duration of the experiments without causing any visible CPE. The continued virus production of RI6318 and LA3331 was similar to that of RV-Dz, but unlike RV-Dz these viruses induced CPE starting at ~14 dpi and resulting in 2-fold reduction of the cell number by 36 dpi. All cells were RV-positive in the infected monolayers at 36 dpi except those infected with CA6944 and OR5810. The fraction of CA6944 infected cells was reduced from ~40% initially to less than 10% at 36 dpi concurrent with 2-log titer reduction. The OR5810 virus production was consistently low (<10^3^ ffu/ml) and only a few infected cells were detected at 36 dpi. Consensus genome sequences of all iVDRVs at 36 dpi were identical to the consensus sequence of the initial inoculums, although minor fractions of different variants would not have been detected by the Sanger sequencing.

The results of these analyses clearly indicate that each iVDRV isolate has unique replicative and persistence properties, which are different from each other and from those of both vaccine and wtRV. Notably, all iVDRV isolates were less cytopathic in cell culture than RA27/3 and had the capability to persist, indicating, at least partially, a wt phenotype in cell culture.

### Neutralization of iVDRVs by sera from MMR vaccinees

**Fig 9:**
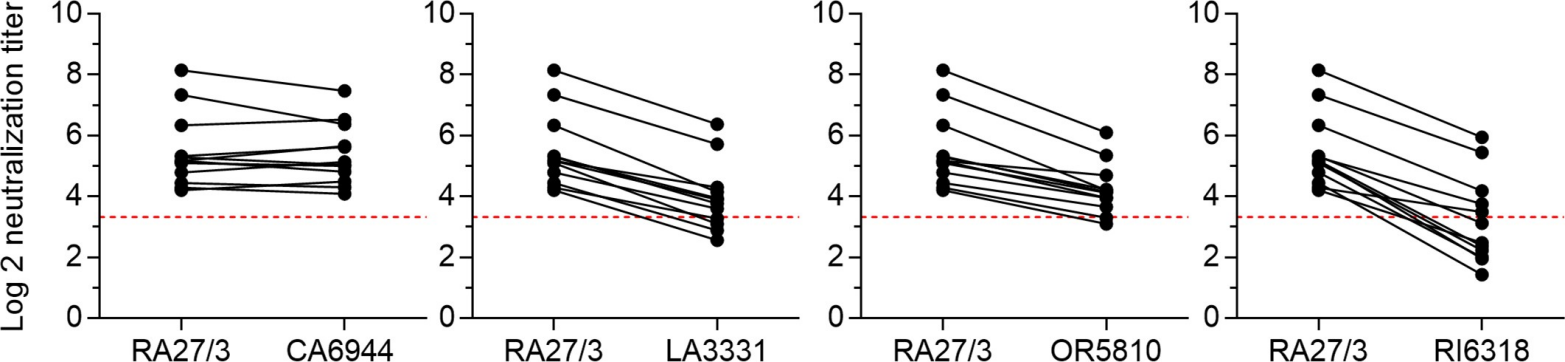
Neutralization of iVDRV isolates by sera from immunized immunologically-competent individuals and PID patients. Comparison of neutralization titers in 10 sera from healthy MMR vaccinees against iVDRVs and RA27/3. A neutralization titer was expressed as a log2 reciprocal of the serum dilution that protected 50% of the input virus. The cutoff of the assay (NT = 10) was depicted by the red dashed line.

Immunization with a RA27/3 vaccine strain protects against infections with different RV genotypes. However, the mutation spectra of iVDRV and wtRV differ substantially (S2 Table). To evaluate the protective capacity of rubella vaccination against iVDRV strains, we compared the neutralization titers in 10 sera from healthy adult vaccinees against RA27/3 and against each of the four iVDRV strains. In general all vaccinee sera contained RV neutralizing antibody that could better neutralize RA27/3 relative to iVDRV. The exception was CA6944 (Fig 9). The RI6318 isolate was the most resistant to neutralization since 7 out of 10 sera failed to effectively neutralize it. These data suggest that additional research on the immune response of vaccinated individuals against iVDRV is warranted.

Next we examined rubella antibody titers and neutralization titers against RA27/3 and iVDRV strains in serum samples from three PID granuloma cases. All case patients were on supplemental immunoglobulin therapy and thus patient’s sera presumably contained a mixture of RV antibodies, both produced by their immune system and infused. The CA and LA case patients, who were on intravenous IgG (IVIG) therapy, had RV IgG titers substantially higher than a typical vaccinee titer (<50 IU/ml) [49], while the RI case patient, who was on intramuscular immunoglobulin therapy (IMIG), had RV IgG titer comparable to that in vaccinees (Table 3). Neutralization titers of all three patients against RA27/3 and three iVDRV strains were substantially higher compared to the typical neutralization titers of less than 100 IU/ml in vaccinated individuals (Table 3). Nonetheless, high levels of neutralizing antibody were not sufficient to eliminate persisting iVDRV in skin. Notably, the serum samples of CA and RI case patients were positive for RV IgM antibody (Table 3), but negative for measles and mumps IgM. The presence of RV IgM antibodies in sera of PID patients long after MMR vaccination and little chance of exposure to wild type rubella in the US may be a marker of ongoing vaccine virus replication and persistence.

**Table 3:** Rubella, measles, and mumps IgM status, rubella IgG and neutralization titers in serum samples of PID patients and MMR vaccinee.

Serum sample	Patient treatment	Measles & mumps IgM	Rubella serology		Neutralization titer against			
			IgM	IgG (IU/ml)	RA27/3	CA6944	LA3331	RI6813
CA case	IVIG	Neg	Pos	6273	>640 ^a^	>640	>640	>640
LA case	IVIG	Neg	Neg	3234	>640	>640	>640	>640
RI case	IMIG	Neg	Pos	52	>640	>640	>640	>640
MMR vaccinee ^b^	none	Neg	Neg	33	94	81	40	8

^a^ The patient sera completely neutralized the indicated viruses at the highest dilution of 1:640 used in the neutralization experiments. The precise titers were not determined.

^b^ Serum from a typical healthy donor.

## Discussion

In this study, we report the isolation of infectious iVDRV strains from cutaneous granulomatous lesions of four PID patients and describe their genetic characteristics, the overall characteristics of their molecular evolution, their replicative properties and their immunologic properties. Our first attempt to recover infectious rubella virus from a skin lesion of a single PID patient was unsuccessful [17]. Successful virus isolations this time may be attributed to a combination of two factors, the use of an optimized virus culture procedure and the presence of less diverged iVDRV strains in the cases described here compared to the previously reported case [17].

One of the key findings of the study is a discovery that all tested granulomas contained infectious iVDRV, which have different biologic properties in tissue culture, compared to the parental vaccine virus. A remarkable feature of the isolated iVDRV is their changes in cytocidal characteristics in tissue culture of cells derived from fibroblasts, presumably an initial target of RA27/3 replication after subcutaneous vaccination. Notably, RA27/3 was cytopathic in fibroblasts whereas wt RV strains were not cytopathic in most cell types tested, including fibroblasts, and establish long-term persistent infections [21, 50]. Unfortunately, no easily available animal model for rubella virus pathology exists and thus extending these tissue culture results will likely require natural history studies in both PID patients and normal individuals vaccinated with RA27/3. Changes in tissue tropism of iVDRVs would be a significant observation, especially if evidence of transmission to normal individuals is found.

Whole genome sequencing revealed that each of the recovered viruses contained multiple predominantly virus-specific nucleotide and amino acid substitutions with respect to RA27/3, which gives a molecular basis for the differences in their biological properties from each other and vaccine virus. Our estimate of the overall evolutionary rate of iVDRV (1.8 x 10^−3^ subs/site/year) based on the whole genome sequences is consistent with evolutionary rates reported for other small RNA viruses [51] and somewhat higher that those reported for wtRV (genotype 1E: 9.16 × 10^−4^–1.04 × 10^−3^ subs/site/year) in populations with person-to-person transmission [52]. Higher iVDRV evolution rates may be due to continuous viral replication and selection during chronic infection in a single individual as opposed to intermittent replication and selection during disease outbreaks.

We have also documented for the first time that iVDRV persisted in a lesion as a diverse population of heterogeneous but closely related quasispecies. Many persisting RNA viruses are maintained in a host as quasispecies [53]. High quasispecies complexity allows viruses to quickly adapt to changing conditions within a host and thus represents a challenge for antiviral therapies, e.g. selection of drug-resistant virus variants. Indeed, mutant spectrum complexity could be a factor in predicting disease progression and response to antivirals [54, 55]. Coexistence of two genetically distinct quasispecies subpopulations in the CA patient granuloma and two iVDRV lineages in the NP cavity and skin lesion of the LA patient which we report here suggests that a viral population structure in chronically infected patients could be more complicated than currently appreciated. Distinct lineages at each site of persistence and the lack of viremia indicate that the virus spreads locally by cell-to-cell spread perhaps because circulating virus (viremia) can be effectively neutralized by the high level of RV neutralizing antibodies detected in the patients. Granulomas are often present in multiple body locations in the skin as well in internal organs, e.g. liver, kidney or spleen [18, 19]. It would be interesting to compare virus sequences from multiple granuloma sites in more patients to verify that multiple iVDRV lineages may persist and evolve independently at different body sites and whether recombination between lineages occurs. Such compartmentalization of genetically and possibly antigenically distinct viral subpopulations, which has been reported for a number of viruses including hepatitis C and HIV, may contribute to the maintenance of persistent infections [56, 57].

The error-prone RdRp is certainly a major contributor in generating sequence diversity in many small RNA viruses [55]. However, our study provides evidence that the cellular factors, one or more tCn-specific APOBECs and ADAR, are the most prominent source of mutations in persisting iVDRV genomes (Fig 6), increasing genetic diversity and thus participating in iVDRV evolution. Interestingly, high levels of expression of the APOBEC3 genes and APOBEC3-mediated C-to-U RNA editing activity of a subset of cellular RNAs have been detected in monocytes and macrophages [33, 58], one of the target cells for RV in granulomas in PID patients [17]. RNA editing by the host ADAR system has been proposed as a factor of evolution of many positive and negative-sense RNA viruses [30, 59], whereas the ability of APOBEC3 family members to restrict viral replication has been reported only for four RNA viruses, measles, mumps, respiratory syncytial virus, and coronavirus [60, 61]. Inhibition of viral replication for these 4 viruses was only demonstrated *in vitro*, in cell cultures overexpressing APOBEC proteins, and was mediated by APOBEC activities other than cytidine deamination. To our knowledge, our report is the first study describing mRNA editing signature of APOBECs in a genome of RNA virus after long-term persistence *in vivo*. However, more studies are needed to assign RV RNA editing activity to specific members of the APOBEC family.

Effective iVDRV population sizes in granulomas are expected to be smaller than those seen in some other viral infections given low quantities of genomic RV RNA in the biopsies and given the small foci of infection consisting only of several infected cells by IHC of PID associated granulomas [17]. Therefore, we expected that persisting iVDRV will undergo sequential genetic bottlenecks when a limited number of founder viruses spreads within a host generating independent virus population at different locations. Linear accumulation of substitutions, both synonymous and nonsynonymous, in iVDRV genomes in different individuals and different body sites over the period of persistence is compatible with the prediction that random genetic drift is the main force driving iVDRV genome evolution, at least during a chronic phase of infection. Another known consequence of genetic drift is an appearance of numerous atypical mutations, which could be the molecular basis of the fitness loss of persisting viruses [55]. Indeed, the majority of amino acid substitutions were specific for iVDRV strains and many of them occurred in the positions that are invariant in wtRV, e.g. mutations in the highly conserved p150 methyltransferase domain and RdRp catalytic domain (S2 Table).

Strong negative selection was observed for nonstructural proteins p150 and p90 as evidenced by the dN/dS ratio of less than 0.2. The structural proteins were less constrained with E1 being more conserved than C or E2. This is consistent with the data obtained for evolution of wtRV in immunologically competent individuals [52, 62]. We have also identified several possible regions under positive selection (mutated B and T cell epitopes, Table 2). Since adaptive evolution of viruses is often strongly coupled with their antigenic evolution, it is reasonable to suggest that the mutations in the predicted B and T-cell epitopes in iVDRV have been positively selected by the combined immune pressure of immunoglobulin therapy and an ongoing cellular and humoral immune response from the patient. Data on poor neutralization of iVDRV strains by some sera from vaccinated individuals further support the hypothesis that the emergence of immune escape variants might be one of the mechanisms of iVDRV persistence. There were, however, discrepancies between the presence of mutations in the E1 neutralizing epitopes and the ability of some iVDRV to avoid neutralization by vaccinees sera. For example, the virus most resistant to neutralization, RI6328, did not have mutations in known E1 epitopes, whereas CA6944 had mutations in 3 out of 4 known epitopes, but was neutralized effectively. It is a likely possibility that not all neutralizing sites in RV have been identified. The number of known CTL epitopes is also small; only three CTL epitopes, all in C protein, have been reported and it is not known to what extent sequence variability in those epitopes can be tolerated [44, 45]. Overall, poor understanding of the antigenic structure of RA27/3 vaccine and wtRV strains hampers investigations of possible mechanisms of iVDRV escape from immune surveillance and its contribution to iVDRV persistence.

One puzzling observation was the detection of extremely high titers of RV neutralizing antibodies in the PID patient sera. The observed lack of viremia in all three tested patients was most likely due to continual removal of iVDRV from the bloodstream by these neutralizing antibodies. It is presently unknown whether the neutralizing antibodies originated from supplemental immunoglobulin therapy or were produced by the immune system of the chronically infected patients. RV neutralization titers (ranging from 40 to 2040) were reported for commercial lots of immunoglobulin produced prior to introduction of rubella vaccine [63]. Determination of RV neutralizing titers in currently produced immunoglobulin preparations, and in sequential serum samples collected at different times after initiation of immunoglobulin therapy, may help to define the contributions of IVIG therapy and a patient’s own immune system to the neutralizing capacity of patient sera. This work would likely enhance our understanding of the role of antibodies in clearance of other RV chronic infections.

In addition to the detection of the high levels of neutralizing antibody, we have also detected RV-specific IgM antibody, but not measles or mumps specific IgM, in the sera of two PID patients. It is unlikely that the RV-specific IgM antibody was from IVIG therapy, since commercial immunoglobulin products contain primarily IgG from the plasma of a thousand or more healthy blood donors with only trace amounts of IgM. Further investigations of RV IgM levels in a larger group of PID patients should be considered to evaluate whether the persistent RV IgM responses long after vaccination may serve as a marker of ongoing RV replication and may predict granuloma formation prior to the development of lesions. Similarly, hepatitis C specific IgM antibody has been observed in patients with chronic hepatitis and their levels correlates with the level of viral replication and treatment outcome [64].

Detection of iVDRV RNA in nasopharyngeal secretions raises a concern about the possible risk of iVDRV to non-immune individuals. Since only a low level of RV RNA was detected in 2 out of 5 sequential NP samples from one case out of three tested, the risk may be low. The frequency and intensity of virus secretion into the NP cavity as well as transmissibility of these viruses to non-immune contacts should be determined in a larger study group. In the US, an estimated 4000 individuals with PID have granuloma complications and thus may be potential iVDRV carriers [15]. Long-term iVDRV secretion by these individuals may have implications for rubella and CRS elimination programs. Even if iVDRV carriers shed low virus quantities, opportunities to transmit virus to susceptible contacts including non-immune siblings and other patients in PID clinics may occur during decades-long iVDRV persistence.

One of the limitations of our study is the use of diagnostic skin samples, one per patient, collected at different times after vaccination, rather than multiple samples from each patient collected serially over time as would be used for a study of pathogen evolution within a patient. A detailed understanding of rubella virus persistence in PID patients will require in part the accumulation of information on the viruses from more patients and from serial samples from individual patients. Unfortunately, the only reliable specimen so far known for iVDRV isolation and sequencing is a skin biopsy. Obtaining sequential skin samples from PID patients, most of them children, over an extended period of time would be challenging and would require human subject oversight appropriate for such a research study. Nonetheless, important conclusions regarding the overall trends of iVDRV evolution within a PID host and properties of persisting iVDRV can be drawn from the analysis of the limited number of diagnostic biopsy samples described here.

We already find it intriguing that most PID patients with RV positive persistent granuloma have defects in their cellular immunity and that we find mutations in T-cell epitopes of viruses from them. Furthermore, T-cell exhaustion is known to be important for other chronic viral infections [65]. We hypothesize that cellular immune defects delay viral clearance and exacerbate what would be a limited persistent infection in persons with normal immunity, leading to years or decades long infections and resulting in damage to multiple tissues in PID individuals. However, we note that limitations in existing data leave open the possibility that other factors, e.g. changing tissue tropism for the virus or antibody escape mutants, are the crucial factors determining this extended persistence. In addition, since the most important property of rubella viruses, their teratogenic potential, is poorly understood, we cannot accurately predict the effect of mutations found here on teratogenic potential from the tissue culture results presented here.

When the disease associations for iVDRV [14, 17] and the sequence variation in iVDRVs described here are compared with other prolonged viral infections in immunocompromised persons, some similarities and some significant differences are found. The number of persons with PID who have prolonged excretion of vaccine-derived poliovirus (iVDPV) is globally only around 100 over the past 54 years and there are only a few case reports of such individuals with disease/death associated with chronic polio vaccine replication [66]. There are thousands of persons currently with cutaneous granulomas associated with iVDRVs predicted to be in the United States alone [15], and the association between RV and granulomatous disease has been established in blinded studies [17]. The median excretion time for prolonged poliovirus in PID is only 1.3 years compared to decades-long RV replication in granuloma. Influenza virus infections in immunocompromised patients, e.g. transplant patients, can become chronic, but only for months, and the virus is usually cleared [67].

Comparisons of sequence divergence between viruses with very different replication properties and genome sequence stability is usually of limited utility; nevertheless some parallels are observed between iVDRV genome evolution and other chronic RNA virus infections. For example, the genomes of poliovirus and influenza virus are also under purifying selection but selection for and against specific changes is found as well. With poliovirus, the accumulation of mutations and genetic rearrangements likely increases the chances of reversion to neurovirulence, but the strains from immunocompromised persons are still vaccine-like [66]. We report here changes in iVDRV in their cytopathologic characteristics, but the lack of an animal model for rubella virus induced pathology limits further evaluation of the significance of these tissue culture findings, in an experimental setting. For influenza virus, the recent report of parallel evolution of viruses in immunocompromised persons and in global circulation is intriguing [68], but since iVDRVs are not known to circulate, important parallels with this report are unlikely. We report differences in the mutations in iVDRV and changes in wild type viruses (S2 Table), but the significance of these differences will require analysis of more sequences. In addition, unfortunately, the molecular surveillance for rubella viruses is far inferior to that for influenza virus or poliovirus, which is also a significant constraint on testing similar parallel evolution of rubella viruses in PID patients.

In conclusion, this study provides the first evidence for the presence of infectious vaccine derived rubella viruses in granulomas of PID patients and evidence for RV genomic RNA in their NP cavities. Our results strongly indicate the ongoing replication and evolution of the rubella vaccine in PID persons resulted in emergence of iVDRV strains with biological properties distinct from RA27/3. To our knowledge, this is the first study detailing mRNA editing signatures associated with APOBEC enzymes in an RNA virus. Furthermore, we provide evidence that most of the iVDRV genome is under negative selection and random genetic drift is the primary mechanism for the evolutions of iVDRV genomes with positive selection playing a role. These results may provide information useful in designing therapies for PID patients with granulomas associated with rubella vaccine. Finally, persistence of rubella virus occurs in a number of serious diseases, e.g. CRS and Fuchs uveitis, and findings from persistent rubella virus vaccine replication in PIDs may be useful in understanding these rubella virus-associated diseases.

## Methods

### Patients and clinical samples

Detailed case descriptions have been published separately [69, 70]. Briefly, all four patients received MMR vaccination at about one year of age and one patient received the second dose at age 5 (the OR case). Three patients were diagnosed with ataxia telangiectasia and one patient had diagnosis of Nijmegen Breakage Syndrome (Table 1). They developed cutaneous granulomatous lesions in different body sites, e.g. face, chest, arms or legs, at various times after vaccination. No pathogenic organisms were identified by the histochemical staining or bacterial, fungal and mycobacterial cultures. The CA and LA case patients were on IVIG therapy and RI case patient received IMIG every 3 weeks. Topical and systemic administration of anti-inflammatory drugs and IgG therapies in all these patients did not result in any significant clinical improvements. The RI case patient was deceased at age 19 years. Types of specimens collected for diagnostic purposes and timing of collection are indicated in Table 1 for each patient. Punch biopsies were taken from the affected skin, immediately snap frozen in liquid nitrogen, and stored at -80°C until testing. The slides cut from the archival formalin-fixes paraffin-embedded (FFPE) tissue blocks of skin biopsies for LA, RI and OR case patients were also submitted to the CDC for testing.

### Ethics statement

Diagnostic samples were obtained after provision of written informed consent (CA, RI, and LA cases) by attending physicians. Verbal consent was obtained from the OR case patient and parent for diagnostic skin biopsy and documented in the patient's chart, which is a standard at Oregon Health & Science University for this minimal risk procedure. The samples were submitted to the Rubella Laboratory (CDC, Atlanta, USA) by the state public health laboratories for molecular testing, virus culture and rubella serology, which were performed as a part of the reference and surveillance responsibilities of the CDC laboratory. Since RV analyses were conducted for the purpose of public health response, this work was determined to not be research in humans by the CDC Institutional Review Board (project determination numbers P_2017_DVD_Icenogle_415 and P_2017_DVD_Icenogle_330). Serum samples from 10 healthy individuals with MMR immunization history were collected by the Emory University donor services with written consent obtained from each participant.

### Cell cultures

Vero cells (ATCC #CCL81) and human primary fetal fibroblasts WI-38 (Coriell Institute, Camden, NJ) were maintained in DMEM supplemented with 5% and 10% Fetal Bovine Serum (FBS, Atlanta Biologicals), respectively.

### Virus culture

Half of each snap-frozen skin biopsy was used for virus culture and the other half for RNA isolation (see below). Skin tissues were homogenized in 0.5 ml of DMEM/2% FBS/ 1% antibiotic-antimycotic (Invitrogen) using zirconium beads as described elsewhere [17]. Tissue homogenate or material from a swab was inoculated into subconfluent Vero cells monolayers in a T12 tissue culture flask, virus was adsorbed for 2 h, and then 3 ml of DMEM/2% FBS was added to the flask. Medium was replaced the next day. Since RV is typically not cytopathic in Vero cells, instead of using our standard protocol for three blind passages of culture media, all cells were transferred from a T12 (passage 1, day 7 (P1-D7)) to a T25 flask after one week of cultivation. Similarly, after an additional week, all cells from a T25 flask (P1-D14) were transferred to a T75 flask and incubated for the third week (P1-D21). This modified virus isolation technique allowed improved recovery of infectious virus from samples containing low amounts of RV. To monitor the amount of infected cells, ~1x10^4^ cells were seeded on chamber slides after each transfer of cells into a larger flask and immunostained for rubella structural proteins using mouse monoclonal antibodies as described previously [48]. Presence of RV RNA in the culture media was monitored by real time RT-qPCR [71, 72]. Depending on the virus concentrations in the media, P1 virus stocks were harvested either at day 14 or day 21.

### Preparation of high titer virus stocks

Preparation of high titer stocks (P4) of RA27/3 and RV-Dz (RVi/Dezhou.CHN/02, genotype 1E) using a bioreactor was described elsewhere [48]. The P1 iVDRV isolates were passaged in Vero cells three additional times using our standard protocol and high titer P4 virus stocks were then prepared by concentrating infected cell media with a Jumbosep 300K Centrifugal Device (PALL Life Sciences) according to the manufacturer's instructions. Viruses were titered on Vero cells and foci of infection were detected by an immunocolorimetric assay [73].

### Rubella, measles and mumps serology

Rubella IgG titers were measured by an enzyme-linked immunosorbent assay (EIA) with the ZEUS ELISA Rubella IgG Test System (ZEUS Scientific, Branchburg, NJ) according to the manufacturer’s instructions and expressed as IU/ml. Rubella IgM testing was performed with a Rubella IgM Capture EIA Kit (Diamedix, Miami, FL). Measles and mumps IgM testing was performed using in-house capture IgM EIA assays [74, 75].

### Virus yield analysis

Vero or WI-38 cells were seeded in 48-well plates at 1x10^5^ cells/well. On the next day the cell monolayers were infected with RV strains at an MOI of 5 ffu/cell or 0.1 ffu/cell (three experiments with two replicate wells per each virus strain). Following 1-hr adsorption at 37°C, unbound virus was washed away with three changes of HBSS and then 0.5 ml of fresh medium was added to the infected monolayers. Virus titers in the medium were determined by titration on Vero cells using immunocolorimetric assay [73]. The cell monolayers were then fixed with 100% methanol, stained for E1 by immunofluorescence assay and the percentage of infected cells was determined as previously described [48].

### Growth curve analysis of persistence

Cell monolayers in 48-well plates were mock infected or infected with RV strains as described above (two experiments with two replicate wells per each virus strain). After virus adsorption and HBSS washes, the initial medium samples (0 hpi) were collected 10 minutes after fresh medium addition and subsequent medium samples were collected at 3-6-day intervals. Virus in the collected medium samples was titered on Vero cells in duplicate. At 36 dpi, phase contrast images were taken with a fluorescent inverted microscope AxioImager.A1 (Zeiss, Oberkochen, Germany). Then the remaining cells in mock infected and infected well were collected by trypsinization and counted using a Scepter cell counter (Millipore, Billerica, MA).

### Fluorescent *in situ* hybridization (RNA-FISH)

WI-38 cells on poly-lysine coated chamber slides (BD Biosciences) were mock infected or infected with RV strains at MOI = 5 (two experiments with two replicate wells per each virus strain). RV genomic RNA was detected with the positive strand-specific probe set using the QuantiGene ViewRNA assay kit (Affymetrix, Cat # QV0096) as previously described [21].

### Histological immunofluorescent staining

The presence of rubella antigen in FFPE tissue sections was detected by histological immunofluorescent staining as described previously [17].

### Rubella neutralization assay

Two-fold serial dilutions of heat-inactivated serum samples were prepared ranging from 1:5 to 1:640. Mixtures of 50 μl of each serum dilution and 50 μl of each virus strain (50–100 ffu) were incubated at 37°C for 1.5 hour and then added to Vero cell monolayers in 48-well plates. Following 1-hr virus adsorption, the cells were overlaid with DMEM (Thermo Fisher)/1% FBS/1% Avicel (FMC BioPolymer; Newark, DE) and incubated at 37°C, 5% CO_2_. Pooled serum samples of a vaccinee from a rubella IgM and IgG Seroconversion Panel (Biomex GmbH, Heidelberg, Germany) served as the positive control for the assay. A serum sample from a donor lacking RV neutralizing antibody was included in each assay as a negative control. Infected cells were immunostained for E1 at 2 dpi (RA27/3), 3 dpi (LA3331 and CA6944), 4 dpi (RI6318) or 6 dpi (OR5810) as described [73]. Foci of infection were counted by using an ELISPOT analyzer (CTL; Cleveland, OH). The neutralization titer of a serum sample expressed as the reciprocal of the dilution of the serum that neutralized 50% of added virus was calculated by a dose-response regression analysis using Graph-Pad Prism software v.6.0 (GraphPad Software, Inc., San Diego, CA).

### Molecular analyses

RNA was isolated from snap frozen skin samples using an RNeasy Fibrous Tissue Mini Kit (Qiagen) and from swab samples or culture media using QIAamp Viral RNA Mini kit (Qiagen) according to the manufacturer's instructions. Quantitative real-time RT-qPCR for rubella, measles and mumps viruses, have been described elsewhere [71] [74] [76]. A detailed description of the full-genome sequencing strategy for rubella virus has been published [62]. Briefly, five overlapping genomic fragments were produced by RT-PCR using primers listed in Table 4. The PCR fragments were sequenced by the Sanger method. The sequences of genomic ends were determined using the 5’/3’ RACE kit as directed by the manufacturer (Roche Diagnostics, Mannheim, Germany). The sequences were named according to the WHO RV naming convention [77] with modifications. The RVs prefix was used for the designation of sequences obtained from total RNA isolated from a clinical sample, while the RVi prefix was used for the designation of sequences obtained from recovered RV isolates; epi week in the name indicated the date of sample collection instead of an onset date; the addition of GR or NP indicated that the sequences originated from granuloma and NP swab, respectively. The sequences were aligned using the Muscle algorithm [78] and alignments were annotated according to the RA27/3 genome using Geneious software version 11.1.2 (Biomatters LTD). Phylogenetic analyses were performed with Mega7 software [79]. The number of synonymous substitutions per synonymous site (dS) and nonsynonymous substitution per nonsynonymous site (dN), the dN/dS ratios and the average behavior of each codon for synonymous and nonsynonymous mutations were calculated for all pairwise comparisons of iVDRV RVs sequences with the RA27/3 ancestor sequence by the method of Nei and Gojobory [80] using Synonymous Non-synonymous Analysis Program SNAP v2.1.1 [81] (https://www.hiv.lanl.gov/content/sequence/SNAP/SNAP.html). This program adjusts for multiple hits by using the Jukes-Cantor correction [82].

**Table 4:** Primers used for amplification of whole genomes of RV.

Genomic fragment	Genome region	Sequence (5' to 3')		Fragment size, bp
		Forward primer	Reverse primer	
I	1–3372	CAA TGG GAG CTA TCG GAC CTC G	CAG CGC CAC ATG TCA CTC CCG CAC A	3372
II	1729–4589	CAC YGT GCT CTA CCG CCA CCC	CGC GCG AGA AGG CGA GGT GAA GGT CGA C	2861
III	3683–7640	CCC CTC GAC CCC CTG ATG G	GGT GCC AGT CGC TCA GGT TGT A	3958
IV	6430–9674	CTC TCC ACG ACC CTG ACA C	AGT AYA AGC ATT TGG CAC AGC A	3270
V	8717–9762	ACC GTC AGG GTC AAG TTC CA	20 Ts YTA TRC AGC A	1045

### Molecular coning and Sanger sequencing for quasispecies analysis

An 904-nt fragment of the E1 gene (nt 8672–9577 in the RV genome) was amplified from RNA isolated from a clinical sample or viral stock using the RV genotyping primers [71] and Platinum SuperFi DNA Polymerase (> 100x Taq fidelity, Thermo Fisher). PCR fragments were cloned into pCR-Blunt II-TOPO vector (Thermo Fisher). Plasmid DNA was isolated using a ZymoPure Plasmid Miniprep Kit (Zymo Research) and the plasmid inserts were sequenced with the M13 forward and reverse primers and the RV primers [71]. Within-subject pairwise genetic distances between individual quasispecies were computed using the Maximum Composite Likelihood method with Mega7.

### GenBank accession numbers

Complete sequences of iVDRV genomes have been deposited in the NCBI database under accession numbers MK787188—MK787191 and MK780807- MK780812.

### 3D structure analysis of E1 substitutions

The structures of the rubella E1 protein (PDB entries 4ADG, 4ADI and 4ADJ) were used as templates for computer modeling. The mutations were modeled as described earlier using the software package ECMMS (Energy Calculations for Macro Molecular Systems) for molecular mechanics modeling [83, 84]. The illustrations of protein structures were prepared using DS Visualizer (http://accelrys.com/products/collaborative-science/biovia-discovery-studio/visualization.html; Accelrys Inc., CA).

### Mutation signature analysis

We first created the list of 993 (out of 1074) mutations, in which ambiguous nucleotides were excluded from the analysis (S1 Data). These mutations were used to evaluate enrichment of APOBEC mutation signatures and the prediction scores for ADAR activity. Enrichment with a mutation signature was defined as the fold excess of actual mutation counts in cytosines of the RV plus strand falling into a signature motif divided by the counts expected with random mutagenesis and calculations were described in detail in [37]. Briefly, we calculated the ratio of two ratios, the ratio of the number of mutations in cytosines falling into signature-specific trinucleotide context to the total number of mutated cytosines as well as the ratio of the number of signature specific trinucleotides to the total number of cytosines in the genomic background. For this background, we considered only the areas of 41 nucleotides centered around the mutated base (column “CONTEXT(+/-20)” in S1 Data Supplementary table; note that this Table displays bases in DNA format to allow compatibility and comparisons with mutation signature outputs of APOBEC mutagenesis in DNA). This allowed us to minimize possible distortion of enrichment calculations by the influence of local RNA secondary structure on mutagenesis, which could excessively shield some genomic areas from APOBEC because this enzyme cannot deaminate cytosines in double stranded structures. The example of the formula to calculate *E*_*uCa*_, Enrichment (i.e., fold-excess over random expectation) for uCa mutation signature is:

$$E_{u\underline{C}a}=\frac{Mut_{u\underline{C}a}\times Con_{C}}{Mut_{\underline{C}}\times Con_{uCa}}$$

where,

*Mut*_*uCa*_−counts of mutations in cytosines falling into uCa motif of the positive strand in a virus isolate

*Con*_*uCa*_−counts of uCa trinucleotide motifs in all 41 nucleotide sequences of the positive strand centered around the mutated positive strand cytosine bases

*Mut*_*C*_−counts of mutations in cytosines of the positive strand in a virus isolate

*Con*_*C*_−counts of C nucleotides in all 41 nucleotide sequences of the positive strand centered around the mutated positive strand cytosine bases.

Unlike the exquisitely specific to single stranded RNA and DNA APOBEC cytidine deaminases, ADARs act on adenines imbedded into double stranded RNA folds, so prediction of ADAR deamination sites should account for combination of knowledge about experimentally defined short nucleotide motifs and a potential to form double stranded structure. The prediction scores for ADAR activity on adenines were generated using the web-based tool InosinePredict (http://hci-bio-app.hci.utah.edu:8081/Bass/InosinePredict) and [85]. A one-sided Mann-Whitney test for the prediction scores testing the hypothesis that scores for [A_to_G] changes characteristic of ADAR would exceed scores for [A–[A_to_G]] nucleotides was performed to obtain P-values shown above the scatter plots.

## Supporting information

S1 Fig RV antigen in chronic granulomatous lesions of LA, OR, and RI case patients. Double immunofluorescent staining of granulomas with M2 macrophage-specific antibodies, CD206 (green), and either RV capsid antibody (Abcam) (red) or measles (MeV) nucleoprotein antibody 83KKII (Millipore) (red, not visible) was performed as described in Methods. Nuclei were counterstained with DAPI. Note strong staining for RV antigen and the lack of staining for measles antigen.
(TIF)

S1 Table Nucleotide (nt) and amino acid (aa) substitutions in iVDRV genomes. (DOCX)

S2 Table Catalog of amino acid substitutions in iVDRV. (XLSX)

S3 Table Rates of synonymous (dS/year) and nonsynonymous (dN/year) substitutions in iVDRV RVs genomes by gene. (DOCX)

S4 Table Mapping of the amino acid substitutions in RVs and RVi variants onto the E1 3D structure. (DOCX)

S1 Data The list of 993 (out of 1074) mutations in iVDRV RVs genomes with unambiguously identified base substitutions. Sequences are shown in DNA format (T instead of U) to maintain compatibility with other outputs of mutation signature R-script.
(XLSX)

S2 Data Alignment of the nonstructural proteins of the 68 wtRV isolates, which circulated worldwide during a period 1961–2012. The alignment was prepared with Mega7.
(MASX)

S3 Data Alignment of the structural proteins of the 68 wtRV isolates, which circulated worldwide during a period 1961–2012. The alignment was prepared with Mega7.
(MASX)

S4 Data The list of pairwise genetic distances between individual quasispecies within primary granuloma sample (RVs) and the P1 CA6944 virus stock (RVi). Genetic distances was computed using the Maximum Composite Likelihood method with Mega7.
(XLSX)

S5 Data The average behavior of each codon for 6 pairwise comparisons to RA27/3 for synonymous and nonsynonymous mutations, by gene. Data for each gene are located in a separate sheet.
(XLSX)

S6 Data RNA editing signatures. (XLSX)
