# Supplementary figures and images for: Infectious vaccine-derived rubella viruses emerge, persist, and evolve in cutaneous granulomas of children with primary immunodeficiencies
Source: PLoS Pathog. 2019 Oct 28;15(10):e1008080. doi: 10.1371/journal.ppat.1008080 (PMC6837625; doi:10.1371/journal.ppat.1008080)

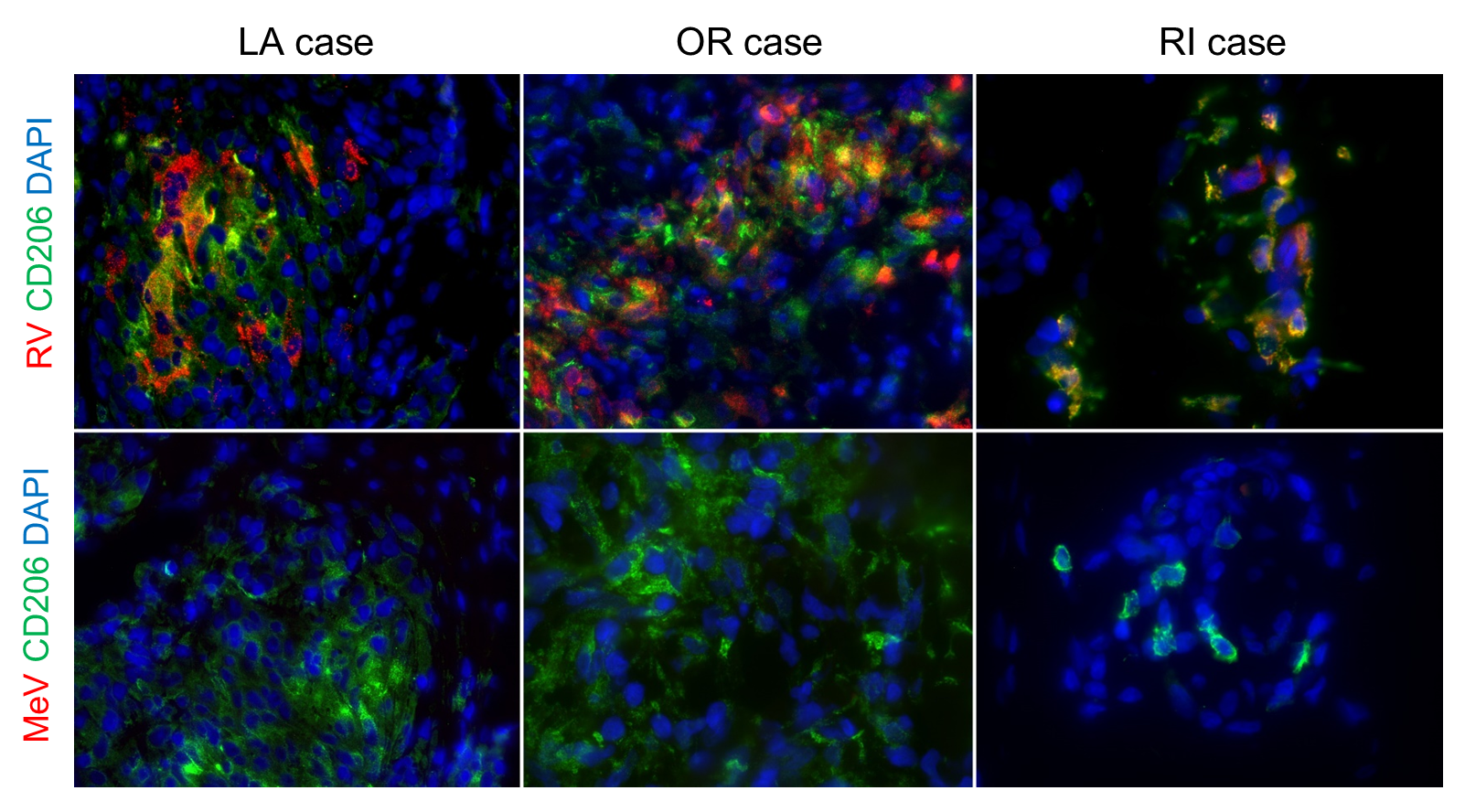

Supplement: S1 Fig — Double immunofluorescent staining of granulomas with M2 macrophage-specific antibodies, CD206 (green), and either RV capsid antibody (Abcam) (red) or measles (MeV) nucleoprotein antibody 83KKII (Millipore) (red, not visible) was performed as described in Methods. Nuclei were counterstained with DAPI. Note strong staining for RV antigen and the lack of staining for measles antigen. (TIF) [file ppat.1008080.s001.TIF]
