# Supplementary material for: Infectious vaccine-derived rubella viruses emerge, persist, and evolve in cutaneous granulomas of children with primary immunodeficiencies
Source: PLoS Pathog. 2019 Oct 28;15(10):e1008080. doi: 10.1371/journal.ppat.1008080 (PMC6837625; doi:10.1371/journal.ppat.1008080)
Supplement: S1 Table — (DOCX) [file ppat.1008080.s002.docx]

**S1 Table.** Nucleotide (nt) and amino acid (aa) substitutions in iVDRV RVs genomes

| **iVDRV** | **Years** | **Number of substitutions** | | | | | | | | | | | | | | |
| --- | --- | --- | --- | --- | --- | --- | --- | --- | --- | --- | --- | --- | --- | --- | --- | --- |
|  |  | **genome** | | **p150** | | **p90** | | **C** | | **E2** | | **E1** | | **5'**  **nt** | **inter-genic nt** | **3' nt** |
|  |  | **nt** | **aa** | **nt** | **aa** | **nt** | **aa** | **nt** | **aa** | **nt** | **aa** | **nt** | **aa** |  |  |  |
| RVs/Oulu.FIN/22.15/GR | 22 | 292 | 69 | 110 | 24 | 78 | 5 | 33 | 12 | 26 | 14 | 41 | 14 | 0 | 4 | 0 |
| RVs/California.USA/43.16/GR | 9 | 95 | 24 | 32 | 6 | 18 | 1 | 6 | 2 | 17 | 6 | 17 | 9 | 0 | 2 | 3 |
| RVs/Oregon.USA/05.18/GR | 11 | 194 | 44 | 75 | 11 | 36 | 6 | 27 | 9 | 24 | 13 | 26 | 5 | 1 | 4 | 1 |
| RVs/RhodeIsland.USA/9.17/GR | 16 | 236 | 60 | 80 | 25 | 48 | 5 | 27 | 9 | 35 | 16 | 39 | 5 | 0 | 4 | 2 |
| RVs/Louisiana.USA/27.17/GR | 5 | 129 | 32 | 51 | 13 | 30 | 3 | 13 | 6 | 14 | 3 | 19 | 7 | 1 | 1 | 0 |
| RVs/Louisiana.USA/27.17/NP | 5 | 128 | 36 | 44 | 9 | 29 | 4 | 24 | 10 | 15 | 6 | 16 | 7 | 0 | 0 | 0 |
