# Supplementary material for: Infectious vaccine-derived rubella viruses emerge, persist, and evolve in cutaneous granulomas of children with primary immunodeficiencies
Source: PLoS Pathog. 2019 Oct 28;15(10):e1008080. doi: 10.1371/journal.ppat.1008080 (PMC6837625; doi:10.1371/journal.ppat.1008080)
Supplement: S3 Table — (DOCX) [file ppat.1008080.s004.docx]

**S3 Table.** Rates of synonymous (dS/year) and nonsynonymous (dN/year) substitutions in iVDRV RVs genomes by gene

|  | **dS** | | | | | **dS/year** | | | | | |
| --- | --- | --- | --- | --- | --- | --- | --- | --- | --- | --- | --- |
|  | **p150** | **p90** | **C** | **E2** | **E1** | **p150** | **p90** | **C** | **E2** | **E1** | **AVR** |
| RVs/Oulu.FIN/22.15/GR | 0.088 | 0.132 | 0.089 | 0.058 | 0.079 | 4.0E-03 | 6.0E-03 | 4.1E-03 | 2.6E-03 | 3.6E-03 | 4.1E-03 |
| RVs/California.USA/43.16/GR | 0.026 | 0.029 | 0.017 | 0.052 | 0.023 | 2.9E-03 | 3.2E-03 | 1.9E-03 | 5.8E-03 | 2.5E-03 | 3.2E-03 |
| RVs/Oregon.USA/05.18/GR | 0.066 | 0.051 | 0.080 | 0.052 | 0.101 | 6.0E-03 | 4.7E-03 | 7.3E-03 | 4.8E-03 | 9.2E-03 | 6.4E-03 |
| RVs/RhodeIsland.USA/9.17/GR | 0.056 | 0.075 | 0.080 | 0.092 | 0.061 | 3.5E-03 | 4.7E-03 | 5.0E-03 | 5.8E-03 | 3.8E-03 | 4.5E-03 |
| RVs/Louisiana.USA/27.17/GR | 0.039 | 0.046 | 0.026 | 0.052 | 0.034 | 7.7E-03 | 9.2E-03 | 5.1E-03 | 1.0E-02 | 6.8E-03 | 7.9E-03 |
| RVs/Louisiana.USA/27.17/NP | 0.035 | 0.041 | 0.057 | 0.043 | 0.025 | 7.1E-03 | 8.2E-03 | 1.1E-02 | 8.5E-03 | 5.1E-03 | 8.0E-03 |
|  |  |  |  |  | **AVR** | 5.2E-03 | 6.0E-03 | 5.8E-03 | 6.3E-03 | 5.2E-03 | **5.7E-03** |

|  | **dN** | | | | | **dN/year** | | | | | |
| --- | --- | --- | --- | --- | --- | --- | --- | --- | --- | --- | --- |
|  | **p150** | **p90** | **C** | **E2** | **E1** | **p150** | **p90** | **C** | **E2** | **E1** | **AVR** |
| RVs/Oulu.FIN/22.15/GR | 0.0089 | 0.0027 | 0.0199 | 0.0226 | 0.0130 | 4.0E-04 | 1.2E-04 | 9.0E-04 | 1.0E-03 | 5.9E-04 | 6.1E-04 |
| RVs/California.USA/43.16/GR | 0.0021 | 0.0005 | 0.003 | 0.0096 | 0.0083 | 2.3E-04 | 5.6E-05 | 3.3E-04 | 1.1E-03 | 9.2E-04 | 5.2E-04 |
| RVs/Oregon.USA/05.18/GR | 0.0038 | 0.0033 | 0.0137 | 0.0210 | 0.0046 | 3.5E-04 | 3.0E-04 | 1.2E-03 | 1.9E-03 | 4.2E-04 | 8.4E-04 |
| RVs/RhodeIsland.USA/9.17/GR | 0.0087 | 0.0027 | 0.0137 | 0.0259 | 0.0046 | 5.4E-04 | 1.7E-04 | 8.6E-04 | 1.6E-03 | 2.9E-04 | 7.0E-04 |
| RVs/Louisiana.USA/27.17/GR | 0.0045 | 0.0016 | 0.0107 | 0.0048 | 0.0065 | 9.0E-04 | 3.2E-04 | 2.1E-03 | 9.6E-04 | 1.3E-03 | 1.1E-03 |
| RVs/Louisiana.USA/27.17/NP | 0.0031 | 0.0027 | 0.0168 | 0.0096 | 0.0065 | 6.2E-04 | 5.4E-04 | 3.4E-03 | 1.9E-03 | 1.3E-03 | 1.5E-03 |
|  |  |  |  |  | **AVR** | 5.1E-04 | 2.5E-04 | 1.5E-03 | 1.4E-03 | 8.0E-04 | **8.9E-04** |
