# Supplementary material for: Infectious vaccine-derived rubella viruses emerge, persist, and evolve in cutaneous granulomas of children with primary immunodeficiencies
Source: PLoS Pathog. 2019 Oct 28;15(10):e1008080. doi: 10.1371/journal.ppat.1008080 (PMC6837625; doi:10.1371/journal.ppat.1008080)
Supplement: S4 Table — (DOCX) [file ppat.1008080.s005.docx]

**S4 Table**. **Mapping of the amino acid substitutions in RVs and RVi variants onto the E1 3D structure.**

| **Amino acid substitutions** | **Changed in** | | **Surface location*** | **Comments** |
| --- | --- | --- | --- | --- |
|  | **RVs** | **RVi** |  |  |
| T5A | - | + |  |  |
| A24V | + | + |  | Shared substitution |
| G25D | + | + | + |  |
| I32V | + | - |  | Hotspot I |
| A34V | + | - | + | Hotspot I |
| I50M | + | + |  | Hotspot II |
| V57A | + | - |  | Hotspot II |
| T68P | + | + |  |  |
| P70S | + | - | + |  |
| F84L | + | + | + | Hotspot II |
| V87I | - | + |  | Hotspot II |
| Y95H | + | - |  |  |
| E118Q | + | + | + | Hotspot II |
| T137A | + | + | + |  |
| S146G | - | + | + |  |
| H152N | + | + |  |  |
| K158R | + | - | + | Hotspot I |
| V174A | - | + | + |  |
| S175L | + | + | + |  |
| H210V | + | - | + |  |
| L245V | + | + |  | NT2 epitope |
| A260V | + | + |  | NT2 epitope |
| A280M | + | + | + | NT4 epitope |
| I280T | + | - | + | NT4 epitope |
| S282A | + | + | + | NT4 epitope |
| V329I | + | - | + |  |
| A333T | + | - | + |  |
| A333V | + | + | + |  |
| A337V | - | + | + |  |
| Q351R | + | + |  | Hotspot I |
| L361V | + | + |  |  |
| K384R | + | - | + |  |
| A388T | + | + | + |  |
| P415S | + | - | + | Hotspot I |
| T429I | + | - |  |  |
| Q437E | + | - | + |  |
| A440V | + | - | + |  |
| A444T | + | - | + |  |
| A445S | + | - | + |  |

*relative solvent accessibility >0.05, probe size 1.4A
